# Screening of Antimicrobial Activities and Lipopeptide Production of Endophytic Bacteria Isolated from Vetiver Roots

**DOI:** 10.3390/microorganisms10020209

**Published:** 2022-01-19

**Authors:** Yuka Munakata, Egon Heuson, Théo Daboudet, Barbara Deracinois, Matthieu Duban, Alain Hehn, François Coutte, Sophie Slezack-Deschaumes

**Affiliations:** 1 UMR 1121, Laboratoire Agronomie et Environnement (LAE), Université de Lorraine—INRAE, F-54000 Nancy, France; yuka.munakata@univ-lorraine.fr (Y.M.); alain.hehn@univ-lorraine.fr (A.H.); 2 Univ. Lille, CNRS, Centrale Lille, Univ. Artois, UMR 8181—UCCS—Unité de Catalyse et Chimie du Solide, F-59000 Lille, France; egon.heuson@centralelille.fr; 3 Université de Lille, UMRt BioEcoAgro 1158-INRAE, Équipe Métabolites Secondaires d’Origine Microbienne, Institut Charles Viollette, F-59000 Lille, France; theo.daboudet@univ-lille.fr (T.D.); barbara.deracinois@univ-lille.fr (B.D.); matthieu.duban@univ-lille.fr (M.D.); francois.coutte@univ-lille.fr (F.C.)

**Keywords:** bacterial endophytes, lipopeptides, antifungal activities, high-throughput screening, *Bacillus subtilis*, *Fusarium* sp.

## Abstract

The exploration of certain microbial resources such as beneficial endophytic microorganisms is considered a promising strategy for the discovery of new antimicrobial compounds for the pharmaceutical industries and agriculture. Thirty-one endophytic bacterial strains affiliated with *Bacillus*, *Janthinobacterium, Yokenella*, *Enterobacter*, *Pseudomonas*, *Serratia*, and *Microbacterium* were previously isolated from vetiver (*Chrysopogon* *zizanioides* (L.) Roberty) roots. These endophytes showed antifungal activity against *Fusarium graminearum* and could be a source of antimicrobial metabolites. In this study, in particular, using high-throughput screening, we analyzed their antagonistic activities and those of their cell-free culture supernatants against three species of *Fusarium* plant pathogens, a bacterial strain of *Escherichia coli*, and a yeast strain of *Saccharomyces cerevisiae*, as well as their capacity to produce lipopeptides. The results showed that the culture supernatants of four strains close to *B. subtilis* species exhibited antimicrobial activities against *Fusarium* species and *E. coli*. Using mass spectrometry analyses, we identified two groups of lipopeptides (surfactins and plipastatins) in their culture supernatants. Whole-genome sequencing confirmed that these bacteria possess NRPS gene clusters for surfactin and plipastatin. In vitro tests confirmed the inhibitory effect of plipastatin alone or in combination with surfactin against the three *Fusarium* species.

## 1. Introduction

Microorganisms are an important resource for the mining of bioactive ingredients, which can then be used as drugs or leading compounds for the pharmaceutical and agrochemical industries [1,2]. For example, a number of bioactive compounds have been characterized in soil-derived microorganisms such as *Streptomyces* sp. [3]. Currently, discovering new bioactive compounds and/or new modes of action appears crucial to control multidrug-resistant microbes, which threaten the sustainable developmental goals set by the United Nations regarding human health and food security. In addition, due to societal expectations for more sustainable agriculture around the world and in Europe in particular, the characterization of bioalternatives to chemical inputs that are assumed to be less toxic to the environment and human health is vital [4,5]. Hence, exploring microbial diversity in less investigated environments could offer opportunities to discover new bioactive compounds and/or new modes of action.

In this context, endophytic microorganisms could be an attractive reservoir [6]. Indeed, endophytic microorganisms that occupy different ecological niches in the internal tissues of the plant are known to produce a high diversity of secondary metabolites, which can have pharmaceutical and agricultural applications [7,8]. In particular, these microorganisms produce several chemical classes of biomolecules with antimicrobial properties, such as ecomycin, kakadumycin, and munubicins [7,9,10].

Among the antimicrobial biomolecules, lipopeptides can be distinguished [11,12]. These biosurfactants can be produced by different microorganisms. For example, bacterial strains affiliated with the *Bacillus* genus were reported to produce a mixture of lipopeptides of the fengycin (plipastatin), iturin, and surfactin families [12]. These antimicrobial compounds are thought to play a key role in the antagonistic activities of these *Bacillus* strains against various fungal phytopathogens or foodborne pathogens [11,13,14,15,16]. In addition to such direct effects, lipopeptides could aid in plant tissue colonization. Finally, some lipopeptides are also known to induce systemic resistance in plants, which is promising for the development of biocontrol solutions that carry a lower risk of resistance emergence [17,18]. To date, lipopeptides have been characterized in only limited numbers of endophytic bacteria [9,10,11,19].

Since the development of whole-genome sequencing, genome mining has become a powerful tool with which to investigate the potential of a strain for the synthesis of new natural compounds [6]. In particular, non-ribosomal peptide synthetase (NRPS) genes involved in the biosynthesis of lipopeptides are considered to be an indicator for the prediction of the biological activities or functions of certain bacteria [20,21]. NRPS operons often consist of regularly arranged modules, where one module is responsible for the adenylation, condensation, and thiolation of one amino acid, leading to an extension of peptide chains. Due to the modular structure of NRPS, homology analysis of predicted NRPS genes identified thanks to bioinformatic tools can help to predict peptide sequences [22]. A database dedicated to non-ribosomal peptides, named NORINE [23], was developed to help to identify such peptides and could therefore be used to assess the potential of endophytic bacteria to produce lipopeptides.

Vetiver (*Chrysopogon zizanioides* (L.) Roberty) is a perennial grass species used for its essential oil, mainly in the production of perfume. In our previous work, we identified 31 endophytic bacterial isolates derived from vetiver roots, showing strong inhibitory activity toward the phytopathogenic fungus *Fusarium graminearum* in in vitro culture [24]. These strains affiliated with *Bacillus, Janthinobacterium, Yokenella, Microbacterium, Pseudomonas*, *Enterobacter*, and *Serratia* genera could be a source of bioactive molecules with antimicrobial activities such as lipopeptides.

In this work, given the large number of bacterial candidates, we developed a new high-throughput workflow for the evaluation of the spectrum of antagonistic activities of these strains against reference yeast, bacterial strains, and phytopathogenic *Fusarium* species, as well as for the detection of lipopeptides that might be responsible for these activities. This workflow involves an automatic liquid handler for antimicrobial activity characterization and sample preparation and two mass spectrometry analysis techniques for molecule identification. Finally, to confirm the accuracy of the mass analysis, the genomes of the best lipopeptide-producing bacterial strains were sequenced and analyzed by bioinformatics, revealing the presence of NRPS gene clusters responsible for the production of antifungal lipopeptides.

## 2. Materials and Methods

### 2.1. Bacterial and Fungal Strains

A collection of 31 endophytic bacterial strains previously isolated from vetiver roots was used [24]. These strains were selected for their growth inhibition activity against *F. graminearum* in in vitro dual cultures and characterized taxonomically (Table 1). They were stored in a 15% glycerol solution at −80 °C. The 16S rDNA sequences of the 31 strains were deposited in GenBank, and the accession numbers are shown in Table 1.

The phytopathogenic fungal strains used in this study were *F. graminearum*, *F. oxysporum*, and *F. culmorum*. The *F. graminearum* strain was kindly provided by IFBM (Institut Français de la Brasserie et de la Malterie), while the other 2 strains were purchased from BCCM/MUCL: *F. oxysporum* f. sp. *radicis-lycopersici* Jarvis & Shoemaker (MUCL 039790) and *F. culmorum* (W.G. smith) Saccardo (MUCL 042823). The fungal strains were sub-cultured on potato dextrose agar (PDA) (OXOID) plates at 28 °C for *F. graminearum* and 25 °C for *F. oxysporum* and *F. culmorum*.

One strain of *Escherichia coli* K12 (ATCC, Manassas, MA, USA) and one strain of *Saccharomyces cerevisiae* DSM1333 (DSMZ, Braunschweig, Germany) were also used. Both were stored at −80 °C.

### 2.2. Dual Culture Test of Selected Vetiver Bacterial Endophytes against F. oxysporum and F. culmorum

Bacterial strains were cultured in 2 mL of nutrient broth (NB) (Sigma Aldrich, St. Louis, MO, USA) inoculated with a single colony for approximately 24 h at 28 °C. Fifty µL of the liquid culture adjusted to an optical density at 600 nm of 1.0 was placed at 2 points 3 cm from an eight-mm-diameter mycelial plug from a 5–10-day-old culture of *F. oxysporum* or *F. culmorum* on PDA at 25 °C, placed at the center of a Petri dish (9 cm in diameter) containing 20 mL of PDA (Sigma-Aldrich, St. Louis, MO, USA). Three replicates were prepared for each bacterial strain. The plates were incubated at 25 °C. The diameters of the fungal colonies were measured at 6 and/or 11 days, according to the radial growth of the fungal species. The inhibition rate (%) was calculated with the following formula: 100 × (1 − dt/dc), where dt is the mean diameter of the *Fusarium* strains in the test plates and dc in the control plates.

### 2.3. Preparation of Supernatant Samples of Bacterial Endophytes

For evaluation of the inhibitory activity of cell-free bacterial supernatants in diffusion assays, bacteria were cultured at 28 °C 180 rpm for 24 h in 2 mL of King’s B (KB, proteose peptone 20 g/L, KH_2_PO_4_ 1.5 g/L, MgSO_4_, (H_2_O)_7_ 1.5 g/L, glycerol 10 mL/L, pH 7.2) or NB (Sigma Aldrich, St. Louis, MO, USA) medium. Bacterial cultures were then centrifuged at 11,000× *g* and 4 °C for 10 min and the supernatant was filtered through a 0.2 µm polyethersulfone membrane (Acrobatic^®^ Syringe Filter with Supor Membrane). KB and NB media were chosen because they are often used for the growth of heterotrophic bacteria such as *Pseudomonas* and *Bacillus* [25,26]. Moreover, KB medium was also studied for the production of antifungal compounds by *Serratia* strains [27].

For mass spectrometry analysis, a single colony of each endophytic bacterial strain was cultured in 2 mL of KB medium at 28 °C 180 rpm for approximately 24 h. Then, 200 µL of the preculture was transferred into 10 mL of KB in 50 mL sterile tubes and cultured under the same conditions. After 24, 48, and 72 h, 1 mL of each culture was placed in 2 mL sterile tubes and the pH was adjusted to approximately 7 by dropping 1 M KOH. The tubes were then centrifuged at 10,000× *g* at 4 °C for 10 min, and 500 µL of the supernatant was collected in new sterile tubes. As a control sample, we used medium treated in the same way. The samples were kept at 4 °C for 4 to 5 days until analysis by MALDI-TOF.

### 2.4. Antagonism Test of Cell-Free Supernatants of the Selected Vetiver Bacterial Endophytes against Fusarium Species

Cell-free supernatants from a bacterial culture on KB or NB were tested against *F. graminearum*, *F. oxysporum*, and *F. culmorum* in 9-cm-diameter dishes containing 20 mL PDA. One hundred µL of the supernatant sample was placed in 8-mm-diameter wells 2 cm distant from the center of the plate. An 8-mm-diameter fungal plug from a 5–10-day-old culture on PDA at 25 °C of one of the 3 *Fusarium* species was inoculated at the center of the plate. The Petri dish was incubated at 28 °C in darkness. Each plate had three different samples and one control sample. The control sample corresponded to a non-inoculated medium filtered through a 0.2 µm membrane. Three independent replicates were prepared. The radii of the fungal colonies were measured after 3 days (*F. graminearum* and *F. culmorum*) and 8 or 11 days (*F. oxysporum*) of incubation at 28 °C. The inhibition rate was calculated with the following formula: Inhibition rate (%) = 100 × (1 − rt/rc). Herein, rt is the radius of test samples, and rc is the radius of the control sample in the same plate as the test samples.

### 2.5. Fully Automated Workflow for Cell-Free Supernatant Screening for Antimicrobial Metabolites

**Figure 1:**
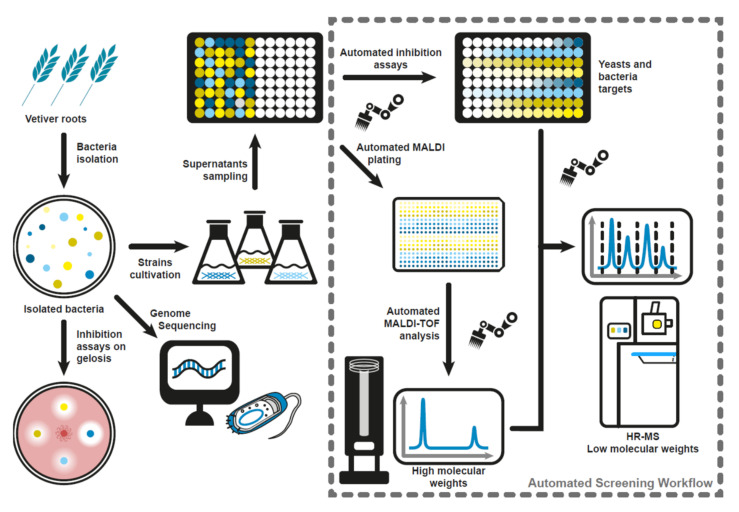
Diagram showing the screening workflow used in this work. This is composed of a first step of screening of antifungal activities on Petri dish and a second robotized step of high-throughput screening of antimicrobial activities and identification of secondary lipopeptide metabolites by high-resolution mass spectrometry.

The workflow used in this study is presented in Figure 1. This workflow requires a liquid handler (Biomek FX^p^, Beckman Coulter, Brea, CA, USA). This robot is used first for the realization of antimicrobial activity in the plates. For this, the cell-free supernatants are placed in contact with the target strains at different dilutions, including the required control experiments, following the plate map described in Figure S1.

The liquid handler was used to first distribute the samples into the different plates required for the liquid assays and MS analysis. This included the direct deposition of the samples onto the MALDI target, after their mixing with the matrix, again using the liquid handler. The plates were then incubated inside an off-robot incubation chamber, and then placed back on the liquid handler for mixing and reading of the plate after the incubation time. Finally, the liquid handler was used to prepare the samples that were analyzed by HPLC-TOF. After incubation at the proper temperature, the prepared plates were read for their OD using a spectrophotometric plate reader (Filtermax F5, Beckman Coulter, 600 nm) present on the robot deck. The liquid handler was also used in parallel for the conditioning of the cell-free supernatants and their automated deposition onto the MALDI target for the first step of mass spectrometry analyses. For this, 15 µL of sample was mixed with 15 µL of matrix (10 mg/mL HCCA matrix in 50/47.5/2.5: acetonitrile/MS-grade water/trifluoroacetic acid) and 2 µL of the mixture was precisely deposited onto each spot of the MALDI target. These analyses were then performed with a matrix-assisted laser desorption/ionization–time-of-flight mass spectrometry (MALDI-TOF, Autoflex Speed, Bruker) to rapidly detect high-molecular-weight secondary metabolites, with a special focus on the lipopeptides that are released by the strains into the culture supernatant. Finally, the samples were gathered into 96-well microtiter plates and then dried using a rotative vacuum concentrator, before being taken in half a volume of 100% methanol. The most promising samples were then analyzed by UPLC-Q-TOF mass spectrometry (Synapt G2Si, Waters) to detect the other potentially produced metabolites and perform high-resolution analyses of the lipopeptides detected with MALDI-TOF. The protocols used for the mass spectrometry analyses are detailed below in their respective paragraphs.

### 2.6. Antagonism Test of Cell-Free Supernatant of the Endophytes against E. coli and S. cerevisiae

*E. coli* and *S. cerevisiae* were precultured in Mueller–Hinton (MH, Sigma-Aldrich, St. Louis, MO, USA) and YPG (yeast extract 10 g/L, bacto-peptone 20 g/L, glycerol 20 mL/L, pH 7.2) media, respectively. The optical density at 600 nm was adjusted to 0.1 before inoculation. The cell-free supernatants (1 mL) from the bacterial cultures in KB medium were transferred manually into a 2 mL DeepWell plate for further use. The supernatant was then mixed with the target strains using a Biomek FXp liquid handler (Beckman Coulter, Pasadena, CA, USA) in a polystyrene 96-well microtiter plate under sterile conditions to achieve the following plate design (Figure S1).

The plates were lidded and then incubated at 37 °C for *E. coli* and 30 °C for *S. cerevisiae* for 24 h. The growth inhibition (GI) was calculated with the following formula: 100 × (1 − (ODt/ODg), where ODt is the mean OD600 value of the test samples and ODg is the mean OD600 value of the growth control samples (MH or YPG + inoculum). For yeast, because filtered KB medium also caused a certain reduction in yeast growth, the inhibition values were normalized to the values of the control samples (KB + YPG + inoculum).

### 2.7. Lipopeptide Detection with MALDI-TOF

For lipopeptide detection in the supernatants, MALDI-TOF mass spectrometry analyses were performed on an Autoflex Speed^TM^ (Bruker Daltonics, Billerica, MA, USA). The molecular mass measurements were performed in the range of 700 to 3500 *m*/*z* and in automatic reflectron mode using FlexControl^TM^ 3.4 software. The equipment parameters were as follows: voltage values of ion sources #1 and #2 set as 19.00 and 16.50 keV, respectively; voltage values of reflectrons #1 and #2 set as 21.00 and 9.50 keV, respectively; lens tension 8.00 keV; pulsed extraction 120 ns; laser intensity between 60 and 70%; smartbeam parameters set to ultra and sample rate; and digitizer settings set to 4.00 GS/s. The MS signals were acquired by summing 10,000 laser shots per spectrum. Prior to each analysis, the spectrometer was calibrated using the monoisotopic values of the manufacturer’s Peptide Calibration Standard II calibrant (Bruker Daltonics, Billerica, MA, USA), containing Bradykinin Fragment 1–7, Angiotensin II, Angiotensin I, Substance P, Bombesin, Renin Substrate, ACTH clip 1–17, ACTH clip 18–39, Somatostatin 28. The calibrant was prepared by mixing 5 µL of calibrant-diluted mixture according to the manufacturer’s specification with 5 µL of a 10 mg/mL HCCA matrix in 50/47.5/2.5: acetonitrile/MS-grade water/trifluoroacetic acid, and 2 µL was then spotted on a Polished Steel 384 MALDI target (Bruker Daltonics, Billerica, MA, USA). Samples were prepared by mixing 5 µL of supernatant with 5 µL of a 10 mg/mL HCCA matrix in 50/47.5/2.5: acetonitrile/MS-grade water/trifluoroacetic acid, and 2 µL was then spotted on a Polished Steel 384 MALDI target (Bruker Daltonics, Billerica, MA, USA). The plate was dried prior to analysis under a laminar airflow hood at room temperature. Mass spectra were visualized using FlexAnalysis software (version 3.4; Bruker Daltonics, Billerica, MA, USA).

### 2.8. Accurate Mass Measurement of Lipopeptides by Liquid Chromatography Coupled to High-Resolution Mass Spectrometry (LC-HRMS)

Ten µL of the collected supernatant was chromatographically separated at 30 °C in reversed-phase ultrahigh-performance liquid chromatography (RP-HPLC) using an Uptisphere Strategy C18-RP column (250 × 3.0 mm, 5 µm particles, Interchim, Montluçon, France) on a biocompatible ACQUITY UPLC system (Waters, Manchester, UK) and an acetonitrile gradient (flow rate 0.6 mL/min) with solvent A (0.1% formic acid/99.9% H_2_O (*v*/*v*)) and solvent B (0.1% formic acid/99.9% acetonitrile (*v*/*v*)) as follows: 5% solvent B for 5 min, then 5 to 100% solvent B for 35 min, followed by washing and equilibrating procedures with 100 and 5% solvent B for 5 and 10 min, respectively. The HPLC eluent was then ionized into the electrospray source of the SYNAPT-G2-Si mass spectrometer (Waters) at a voltage of 3 kV, using a desolvation gas (N_2_) at a flow of 900 L.h-1, a nebulizer gas flow of 6.5 bar, and source and desolvatation temperatures of 120 and 500 °C, respectively. The mass spectrometer was previously calibrated using a sodium formate solution and measurements were performed in sensitivity, positive, and data-dependent modes using the proprietary MassLynx software (version 4.1, Waters). A maximum of 5 precursor ions with an intensity threshold of 1000 were selected for the fragmentation (MSMS) by collision-induced dissociation (CID) with specified voltages ranging from 10 to 15 V and from 20 to 100 V for the lower- and higher-molecular-mass ions, respectively. Mass data were collected in the measurement range of 200 to 2000 *m*/*z* for MS and of 10 to 2000 *m*/*z* for MSMS using lock mass correction with 556.632 *m*/*z*, corresponding to simply charged leucine enkephalin.

### 2.9. DNA Extraction and Whole-Genome Sequencing

The genomic DNA of four endophytic strains affiliated with *B. subtilis* (strains 22, 23, 26, and 28) was extracted using a DNeasy^®^ UltraClean^®^ Microbial Kit (Qiagen, Hilden, Germany).

Genomic DNA libraries were prepared using the Nextera XT Library Prep Kit (Illumina, San Diego, CA, USA) following the manufacturer’s protocol, with the following modifications: input DNA was increased 2-fold, and PCR elongation time was increased to 45 s. DNA quantification and library preparation were carried out on a Hamilton Microlab STAR automated liquid handling system (Hamilton Bonaduz AG, Rapperswil-Jona, Switzerland). Pooled libraries were quantified using the Kapa Biosystems Library Quantification Kit for Illumina. Libraries were sequenced with Illumina sequencers (HiSeq/NovaSeq) using a 250 bp paired-end protocol. Reads were trimmed using Trimmomatic 0.30 with a sliding window quality cutoff of Q15 [28]. De novo assembly was performed on the samples using SPAdes version 3.7 [29], and the contigs were annotated using Prokka 1.11 [30].

The genomes of four *B. subtilis* strains were submitted to antiSMASH (version 6.0.1) [31] to predict the biosynthesis gene clusters of the secondary metabolites.

### 2.10. Effects of Surfactin and Plipastatin on the In Vitro Growth of Fusarium Species 

Surfactin and plipastatin (synonym fengycin) were purchased from Sigma-Aldrich (ref. S3523, SMB00292, respectively). Surfactin was dissolved in dimethyl sulfoxide (DMSO) at 100 mg/mL and then diluted in sterile water to 1 mg/mL. Plipastatin was dissolved in 0.1% DMSO at 1 mg/mL and then filtered through a 0.2 µm pore membrane. A mixture of surfactin and plipastatin was prepared at a ratio = 1:1 (*w*/*w*). The lipopeptides were then diluted to their final concentrations of 5, 10, 25, 50, 100, 200, and 400 µg/mL. As a control, 1, 0.1, and 0.55% DMSO were used for the surfactin, plipastatin, and mixed solutions, respectively. An in vitro assay was performed with the plate layout described in Section 2.4., applying 40 µL lipopeptide solution per well (diameter 6 mm).

### 2.11. Data Analysis

The figures and statistical tests were performed with R studio with R version 3.6.3. To compare the growth inhibition rate of surfactins, plipastatins, and mixed solutions, the Tukey–Kramer test was performed for each concentration, using the multcomp package.

In all the inhibition assays against plant pathogens, *E. coli*, and yeast, the negative inhibition values were replaced with 0%.

## 3. Results

### 3.1. Antifungal Activities of Vetiver Endophytic Bacterial Strains against Plant Pathogens

**Figure 2:**
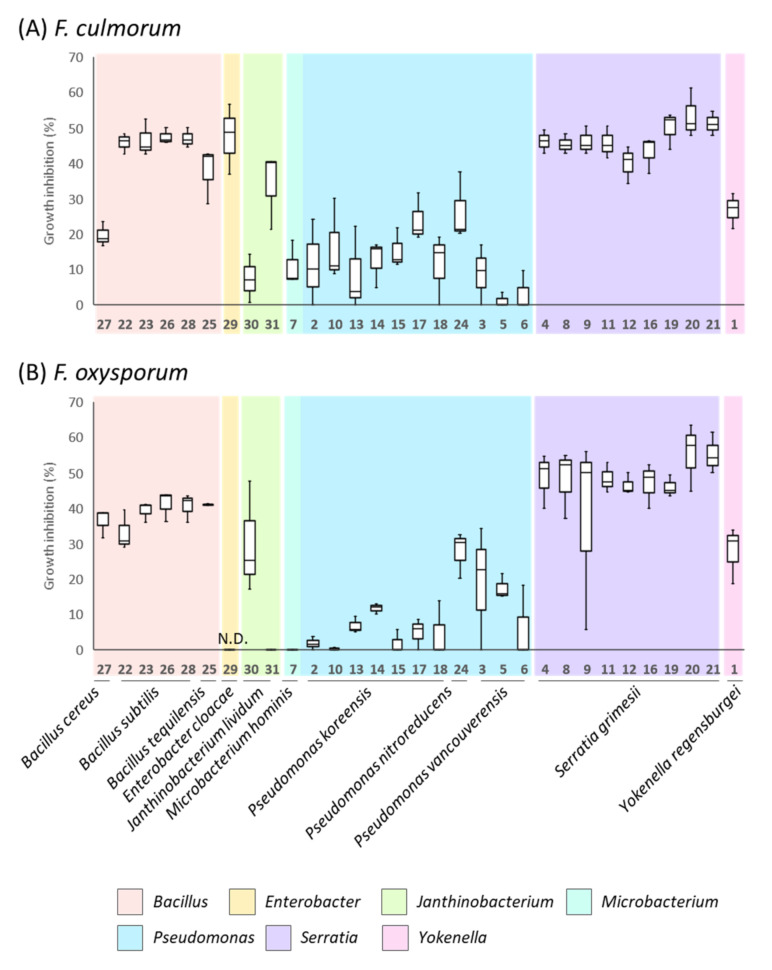
Growth inhibition rate of 31 vetiver bacterial endophytes against *F. culmorum* (Day 6) (**A**) and *F. oxysporum* (Day 11) (**B**).

In this study, 31 selected endophytic bacteria from vetiver roots were investigated (Table S1). These bacteria, affiliated with the *Bacillus*, *Enterobacter*, *Janthinobacterium*, *Pseudomonas*, *Serratia*, *Microbacterium*, and *Yokenella* genera, have previously shown high inhibition rates against *F. graminearum* in *in vitro* cultures (Table S1, [24]). In this study, we evaluated their ability to inhibit the mycelial growth of two other well-known *Fusarium* plant pathogenic species, *F. culmorum* and *F. oxysporum* (Figure 2 and Figure S2). Depending on the bacterial genus, the inhibitory rate varied from 1.1 to 53.4% and 0 to 55.3% against *F. culmorum* and *F. oxysporum*, respectively. Bacteria affiliated with the genera *Serratia* and *Bacillus* showed the highest inhibitory rates against *F. culmorum* and *F. oxysporum* (Figure 2). *Serratia* strains, closely related to *S. grimesii* species, showed similar inhibition rates against *F. culmorum* (approximately 47%) and *F. oxysporum* (approximately 48%). On the other hand, except for the strain affiliated with *B. cereus*, *Bacillus* strains belonging to *B. subtilis* and *B. tequilensis* showed a higher inhibitory rate against *F. culmorum* (approximately 45%) than *F. oxysporum* (approximately 39%). It should be noted that although bacterial strains affiliated with the *Serratia* or *Bacillus* genus showed similar capabilities to inhibit the mycelial growth of *F. culmorum*, the appearance of the fungal colonies differed, depending on the bacterial strains with which the fungus interacted (Figure S2).

For other bacterial strains, the strain affiliated with genus *Enterobacter* had a good inhibitory rate against *F. culmorum* (47%), while bacteria affiliated with *Janthinobacterium, Pseudomonas, Yokenella,* and *Microbacterium* genera showed overall lower inhibitory rates against *F. culmorum* and *F. oxysporum*.

### 3.2. Antimicrobial Activities of the Cell-Free Crude Supernatants of the Vetiver Bacterial Endophytes

The antimicrobial (antifungal and antibacterial) activities of filtered culture supernatants from the endophytic bacteria were then evaluated. For this purpose, KB and NB media were used to grow the bacteria. 

**Figure 3:**
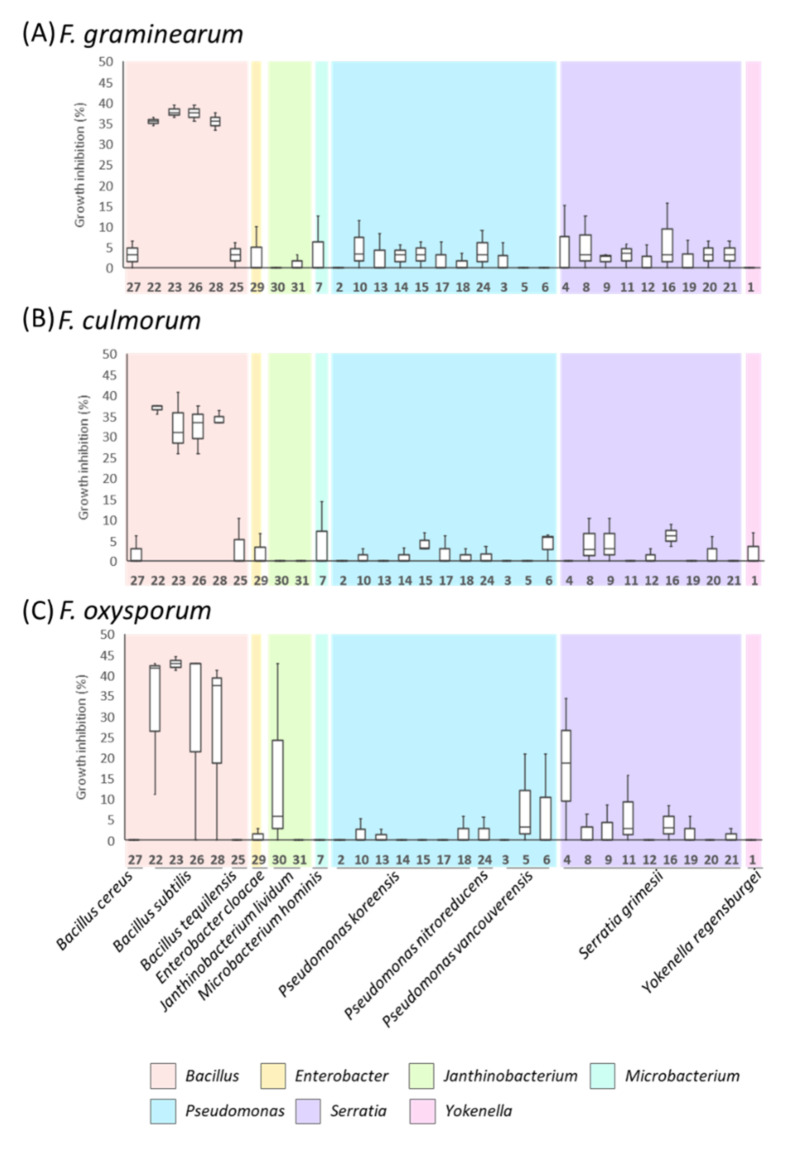
Growth inhibition rates of the cell-free supernatants of vetiver endophytic bacteria grown in KB medium against *F. graminearum* (**A**), *F. culmorum* (**B**), and *F. oxysporum* (**C**).

The inhibitory activities of the bacterial supernatants against *Fusarium* species were tested by diffusion assays (Figure 3). The results obtained showed contrasting inhibitory effects of the culture supernatants depending on the bacterial strains, with inhibition rates ranging from 0% to almost 43% (Figure 3, Table S1). Only supernatants from strains affiliated with *B. subtilis* (strain numbers 22, 23, 26, and 28) and, specifically, those from liquid culture in KB medium displayed a significant inhibitory rate against the three *Fusarium* pathogenic species (Figure 3, Table S1). The inhibitory effects of the supernatants from bacterial culture in NB medium varied depending on the *B. subtilis* strain and the *Fusarium* species (Figure S3).

The inhibitory activities of supernatants of the bacterial cultures in KB medium were also tested against *E. coli* and *S. cerevisiae* in 96-well plates. The supernatants were serially diluted 2 to 8 times. For *E. coli*, only three strains affiliated with *Enterobacter* (strain number 29) and *Bacillus* (strains number 26 and 28) showed little to moderate antibacterial activity (Table 1, Figure S4). The *Enterobacter*-affiliated strain showed the highest inhibition rate (37.6%, observed at the 2-fold dilution), followed by the two *B. subtilis*-affiliated strains, which showed inhibition rates of 16 and 10% at the 8-fold dilution, respectively (Figure S4). Regarding the test with *S. cerevisiae*, no strain showed a growth inhibition effect (Figure S4, Table 1).

### 3.3. Screening for Lipopeptides in Supernatant Samples of Vetiver Endophytes by MALDI-TOF

**Figure 4:**
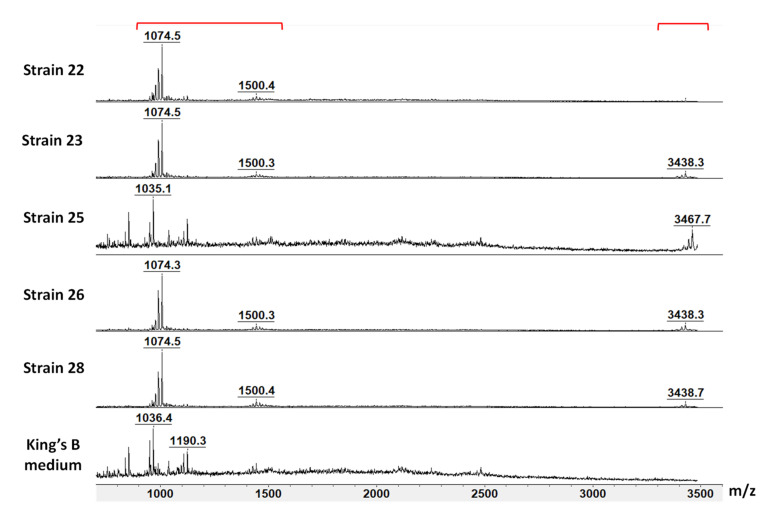
MALDI-TOF mass spectra of supernatant samples of *B. subtilis* (strain 22, 23, 26, 28), *B. tequilensis* (strain 25), and KB medium after 24 h cultures. Mass spectra represent ion intensity in the mass range of 800 to 3500 *m*/*z*. Brackets highlight the two groups of peaks detected.

In a preliminary approach, to screen lipopeptide production by endophytic bacteria, cell-free supernatants from 24 h cultures of the 31 endophytic strains were analyzed using MALDI-TOF, and the main results are presented in Figure 4. KB medium was used as a control and subjected to MALDI-TOF analysis to remove molecules derived from the medium. The peaks at *m*/*z* = 909.0, 925.0, 1019.0, 1020.8, 1034.9, 1036.4, 1173.1, 1188.9, and 1190.3 were, therefore, eliminated. Of the 31 strains analyzed, five belonging to *the Bacillus* genus yielded interesting profiles, as shown in Figure 4. Four strains affiliated *with B. subtilis* (strain numbers 22, 23, 26, and 28) and whose supernatants showed significant antifungal activities against *Fusarium* species had similar peak profiles within the mass ranges of *m*/*z* = 1020 to 1550 (Figure 4). The mass spectra of these strains showed two distinct groups of peaks within the mass ranges of *m*/*z* = 1020 to 1080 and *m*/*z* = 1450 to 1550. The appearance of this group of peaks appears to be similar to that of lipopeptides. Furthermore, we observed that 3 of the 4 strains affiliated with *B. subtilis* (strain numbers 23, 26, 28) and the strain affiliated with *B. tequilensis* (strain number 25) also shared a group of peaks in the mass range of *m*/*z* 3400 to 3480. The latter mass seems to correspond to the production of subtilosin A, a bacteriocin produced by certain strains of *Bacillus*.

In-depth analysis of the culture supernatants at 48 and 72 h was therefore undertaken for these five strains. Compared to strains affiliated with *B. subtilis* (strain numbers 22, 23, 26, and 28), some peaks (such as the ones at *m*/*z* = 1030, 1088, 1485, and 1515) were not detected or were found only after 72 h of cultivation (peaks at *m*/*z* =1044 to 1074) in the supernatant of the strain belonging to *B. tequilensis* (strain number 25) (Table 2). The detected peaks were considered to potentially correspond to two lipopeptide families, surfactins and plipastatins.

The peaks observed in the supernatants of all *Bacillus* strains after 72 h of culture at *m*/*z* = 1030.72, 1044.74, and 1058.75 differed by 14 Da, suggesting a group of compounds with different fatty chain lengths. These peaks were attributed to sodium adducts of surfactin, with fatty acid chains of C13, C14, and C15, respectively (Table 2). As this analysis did not allow us to identify the amino acid at position 7 of the peptidic cycle, it is also possible that these masses correspond to the C14, C15, and C16 isoforms of surfactin A with a valine at position 7. Moreover, the peaks at *m*/*z* = 1060.71, 1074.74, and 1088.73 also differed by 14 Da and were attributed to potassium adducts of surfactin C14, C15, and C16 or to the C14, C15, and C16 isoforms of surfactin A with a valine at position 7. These compounds were found in the supernatants of strains 22, 23, 26, and 28 related to *B. subtilis* after 48 h of culture, whereas they were only detected after 72 h of culture in strain 25 belonging to *B. tequilensis*.

The peaks observed at *m*/*z* = 724.44, 746.43, and 762.42 were identified as the dicharged form of plipastatin A C15 or plipastatin B C13 with hydrogen, sodium, and potassium adducts, respectively. The peaks at *m*/*z* 781.35 were identified as the dicharged form of the plipastatin A C16 or plipastatin B C18 isoform with unsaturated fatty acid chains and sodium adducts. All plipastatin isoforms were observed in the supernatants of the five different strains at 48 and 72 h of culture time. The observed peaks at *m*/*z* = 1485.86, 1515.89, and 1571.63 might be assigned to potassium or sodium adducts of plipastatin A or B (Table 2). The peak at *m*/*z* 1485.86 was only detected after 72 h of culture in the supernatants of strain numbers 22, 26, and 28 (*B. subtilis*). The peak at *m*/*z* 1515.89 appeared only in the supernatants of strains 22, 23, and 28. Finally, the peak at *m*/*z* 1571.63, attributed to a potassium adduct of plipastatin B C19, was only detected in the supernatants of strain numbers 23, 25, and 28 after 48 h of culture and after both 48 and 72 h of culture in the supernatant of strain number 26.

### 3.4. Accurate Mass Measurement by LC-HRMS

**Figure 5:**
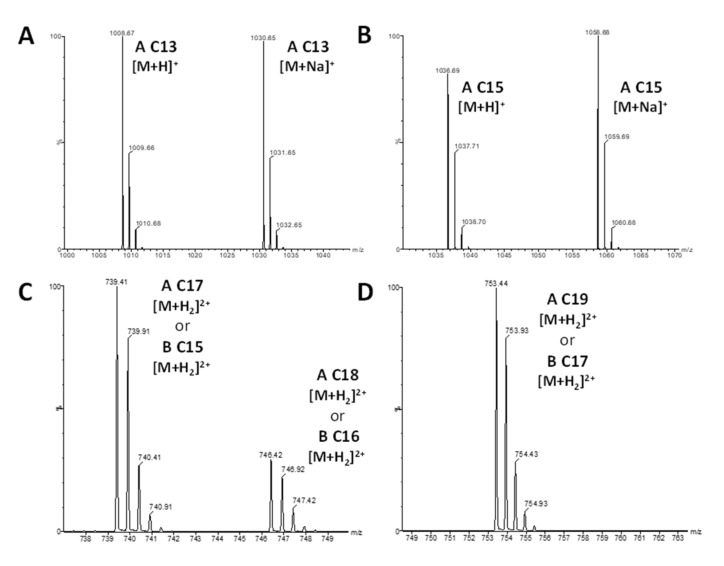
Mass spectra of surfactin (**A**,**B**) and plipastatin (**C**,**D**) from LC-HRMS analysis of supernatant sample of *B. subtilis* (strain 23) after 48 h culture in KB medium.

**Figure 6:**
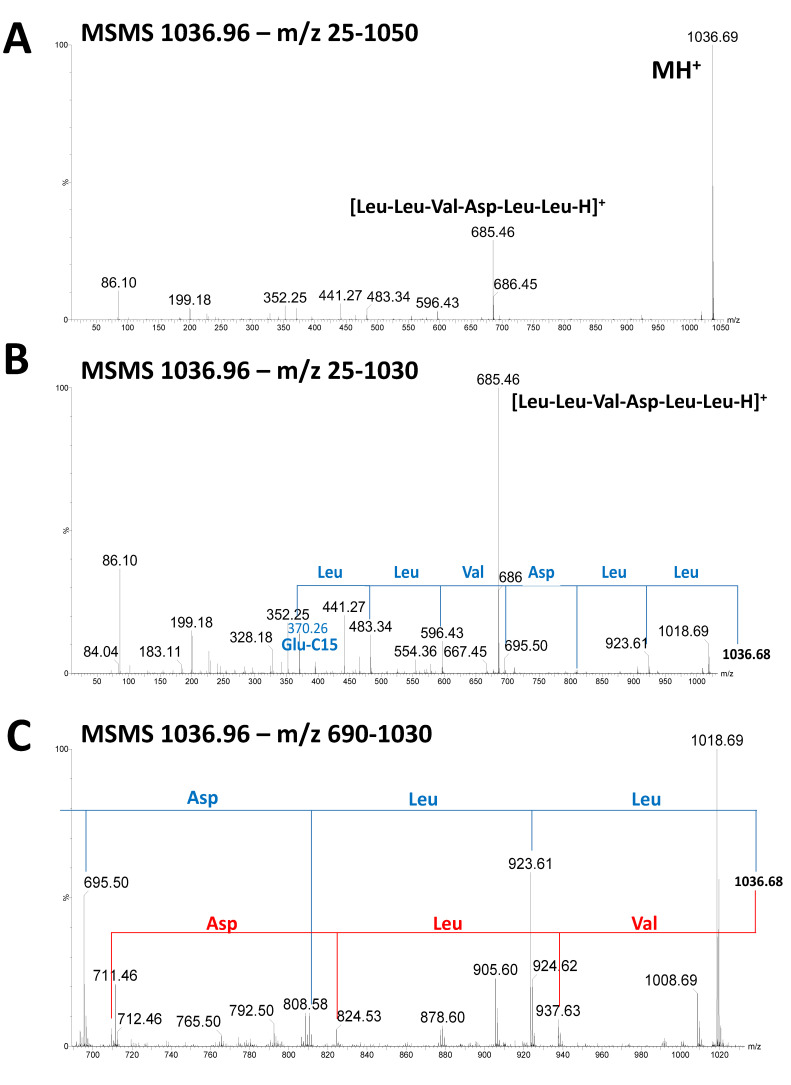
Fragmentation spectra of ion *m*/*z* 1036.96 from LC-HRMS analysis of supernatant sample of *B. subtilis* (strain 23) after 48 h culture. (**A**) corresponds to the full MSMS spectrum, (**B**) to the MSMS spectrum enlarged to the *m*/*z* range 25–1030, and (**C**) to the MSMS spectrum enlarged to the *m*/*z* range 690–1030. The fragmentation proposals for surfactin A C15 and B C16 are presented in blue and red, respectively.

The culture supernatants of the five *Bacillus* strains (22, 23, 25, 26, and 28) were subjected to LC-HRMS analysis to obtain more resolutive mass signals and to assign the correct masses to the lipopeptides. Different masses of surfactins and plipastatins were clearly detected in the five *Bacillus* strains, confirming the assignments proposed in Table 2. Figure 5 represents, as an example of the results, the isotopic clusters of surfactin A C13 (Figure 5A) and C15 (Figure 5B) and plipastatin A C17 or B C15 and A C18 or B C16 (Figure 5C) and A C19 or B C17 (Figure 5D) extracted from the analysis of supernatant of the *B. subtilis* strain 23. MSMS analysis of the different forms of surfactin confirmed the predominant presence of surfactin A C15 with leucine at position 7 (Figure 6, fragmentation pattern in blue). However, MSMS spectra revealed the presence, with a lower intensity, of surfactin B C16 with valine at position 7 (Figure 6, fragmentation pattern in red). Regarding plipastatin, MSMS analysis revealed no informative spectra due to the poor ionization of the different forms of this lipopeptide.

### 3.5. Genome Sequencing and NRPS Cluster Characterization

**Figure 7:**
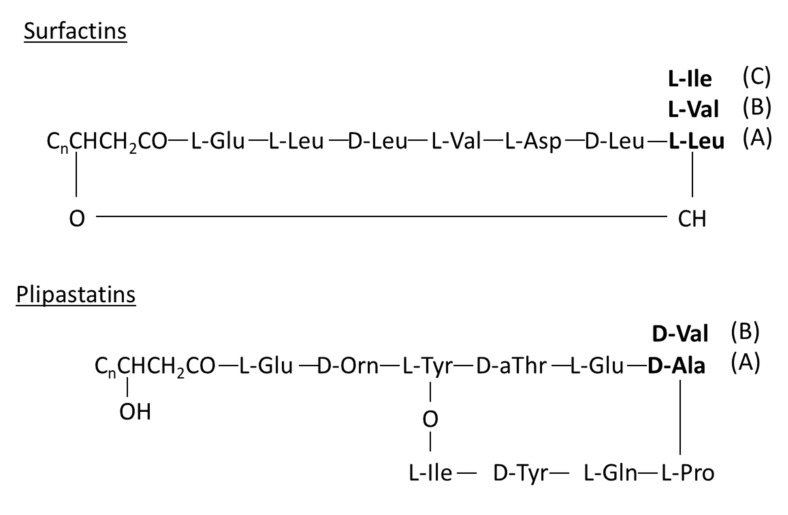
Primary structures of surfactins and plipastatins [11,32]. The letters in brackets correspond to the different possible amino acids in position 8.

Strains affiliated with *Bacillus* and closely related to *B. subtilis* were sequenced. Analysis of draft genomes of the four sequenced *Bacillus* strains showed that these genomes are composed of 4,042,990 to 4,269,420 bp, included in 14 to 26 contigs, with L50 ranging from 377,686 to 2,301,381 bp. GC content ranged from 43.2 to 43.7%. Draft genome annotation identified 4036 to 4338 coding sequences and 83 to 84 tRNA genes. Potential secondary metabolite clusters were screened using the genome mining tool antiSMASH. The *Bacillus* strain close to the *B. tequilensis* species (strain 25) was excluded from this analysis as the culture supernatant of this strain did not show any antifungal activity against *Fusarium* sp. The results showed that the genomes of the four strains might harbor four NRPS gene clusters (Table 3). These predicted clusters included genes encoding lipopeptides, surfactin, and plipastatin (Figure 7), already detected in the supernatants. Notably, the known peptide sequences of plipastatins have glutamine at the eighth position, while antiSMASH affiliated glutamic acid at this position of the putative plipastatin B (Table 3). The difference between glutamine and glutamic acid gives an additional one to the monoisotopic mass of known plipastatin B. The masses obtained during the HRMS analysis are precise enough to confirm the presence of glutamine at position 8 of the plipastatin molecule. Concerning the surfactin, both isoforms with leucine and valine at position 7 are produced by *Bacillus* strains belonging to *B. subtilis* species.

The other clusters might encode the antimicrobial dipeptide bacilysin, the siderophore bacillibactin, and the bacteriocin subtilosin A.

### 3.6. Antagonistic Activities of Surfactins and Plipastatins against Fusarium Species

We analyzed the effects of surfactin and plipastatin identified in the supernatant samples of *B. subtilis* (strain numbers 22, 23, 26, and 28) and *B. tequilensis* strains (strain number 25) alone or combined against *F. graminearum*, *F. culmorum*, and *F. oxysporum.* Surfactin had no clear inhibitory activity against the *in vitro* mycelial growth of *F. graminearum* and *F. culmorum* at any concentration (Figure S5). In contrast, plipastatin exhibited antifungal activities from the 25 µg/mL concentrations for the two *Fusarium* species (Figure S5). Thus, for *F. graminearum*, plipastatin showed an inhibition rate of approximately 15% at a concentration of 25 µg/mL, which increased to approximately 30% for higher concentrations. For *F. culmorum*, plipastatin displayed better antifungal activity, with an inhibition rate of approximately 40% from the concentration of 25 µg/mL. For *F. oxysporum*, inhibition effects were not clear up to concentrations of 100 µg/mL for all solutions. In contrast, plipastatin showed an inhibition rate of approximately 40% from the concentration of 200 µg/mL (Figure S5).

Higher antifungal activities were recorded when plipastatin was tested in combination with surfactin, with growth inhibition of *F. graminearum and F. culmorum* observed from 10 µg/mL. These effects were not observed with *F. oxysporum* (Figure S5).

## 4. Discussion

There is an increasing need to develop original biocontrol solutions in agriculture and to identify new drugs for use as pharmaceuticals to address various social concerns. Microorganisms and/or the associated bioactive compounds that they can synthesize might constitute a promising reservoir for this purpose.

In the present study, we investigated in greater detail the potential of 31 endophytic bacterial strains isolated from vetiver, which showed strong growth inhibitory activity against *F. graminearum* [24]. To better investigate the potential of these strains, we set up an automated high-throughput screening workflow combining antimicrobial testing on microtiter plates and mass spectrometry analysis using MALDI-TOF and LC-HRMS. Thus, these various bacteria were characterized for their antimicrobial activities against microorganisms, including phytopathogenic fungi affiliated with the genus *Fusarium*, yeast, and bacteria. They were also analyzed for their ability to produce lipopeptides that might be involved in any antagonistic activities.

The results that we obtained showed that strains affiliated with *S. grimosii*, *B. subtilis,* and *B. tequilensis* displayed the strongest growth inhibition in an in vitro dual culture against *F. culmorum* and *F. oxysporum*. These results confirm that *S. grimosii*, *B. subtilis,* and *B. tequilensis* strains are useful antagonists of plant fungal pathogens [27,33]. Their antagonistic activities are known to be related to the synthesis and release of a broad range of secondary metabolites with antimicrobial activities. Analysis of the antimicrobial activities of the cell-free culture supernatants of these bacterial strains showed that only the supernatants of *B. subtilis* (strain numbers 22, 23, 26, 28) retained antagonistic activity toward *Fusarium* species but also against *E. coli*. These results may suggest different antibiosis mechanisms between *Serratia* and *Bacillus* strains, whose metabolism is highly variable, especially with regard to the type of antimicrobial compounds produced (soluble and/or volatile compounds) [34]. It should also be noted that the culture supernatants were obtained from mono-cultivated bacteria under non-limiting resource conditions. As a result, some of the compounds involved in the antagonistic activities observed in dual cultures may not have been produced or produced only weakly. Indeed, it has been shown that resource competition and inter- and intraspecific microbial interactions are often implicated in the induction of the biosynthesis of antimicrobial compounds [35,36,37]. 

By using MALDI-TOF analyses, we characterized inhibitory compounds in the cell-free supernatants of *Bacillus* strains. We highlighted the very probable mass of two lipopeptide families. Surfactins and plipastatins were detected in supernatants of the four strains belonging to *B. subtilis* (strain numbers 22, 23, 26, 28) and the strain belonging to *B. tequilensis* (strain number 25). These results are consistent with the presence of NRPS gene clusters detected in the genomes of *Bacillus subtilis* strains. Both molecules are already described to be produced by *B. subtilis* species and the closely related species *B. tequilensis* [38]. Although surfactins are rarely reported as antibacterial molecules except against *Legionella* species [39,40], they could also have antifungal activities and act synergistically with plipastatins (synonyms of fengycins) to potentiate the antagonistic activities of some *Bacillus* strains against phytopathogenic fungi such as *F. graminearum* or *F. oxysporum* [41,42]. In our study, these molecules seem to explain the antagonistic effects of the *B. subtilis* strains toward the three phytopathogenic *Fusarium* species. Other lipopeptides, such as iturins and bacillomycin, have been thought to play a role in the antagonistic activities of some *Bacillus* strains against *Fusarium* species [11,14,43]. In our case, these compounds were not detected in the supernatant samples of *B. subtilis* strains (strain numbers 22, 23, 26, 28) by MALDI-TOF, LC-HRMS, or by whole-genome analysis. This reflects the metabolic variations between bacterial strains belonging to the same species that may result from the different habitats in which these microorganisms have grown before being isolated. 

Finally, it is interesting to mention that our *B. subtilis* and *B. tequilensis* strains, except for *B. subtilis* strain number 22, showed similar MS peak profiles at *m*/*z* over 3000. These compounds could correspond to some bacteriocins, such as a subtilosin A or a variant of subtilosin, which have a molecular weight at *m*/*z* of approximately 3400. Our chromatogram showed minor species at *m*/*z* 3438 that correspond to potassium adducts, as previously described [44,45]. These compounds could explain the activity of *B. subtilis* strains against *E. coli*.

## 5. Conclusions

This paper revealed that four *B. subtilis* strains isolated from vetiver roots originating from Reunion Island have interesting antagonistic activities against several *Fusarium* plant pathogenic species in *in vitro* cultures. These inhibitory activities could partly be due to the production of lipopeptides, surfactins, and plipastatins. These strains are promising for the protection of plants against *Fusarium* diseases. Further studies are now needed to evaluate the crop protection activity of the four *B. subtilis* strains and to study their mode of action *in planta*. It would also be interesting to analyze, for the other strains in this study, such as those affiliated with *Serratia* or *Pseudomonas*, the metabolic traits that may participate in their antagonistic activities in in vitro dual cultures.

## Figures and Tables

**Table 1:** Summary of the in vitro growth inhibition activities of the cell-free supernatants of the 31 vetiver bacterial endophytes against *Escherichia coli* and *Saccharomyces cerevisiae*.

				Cell-Free Supernatant *^1^	
Strain	Genbank Accession	Accession (Munakata et al., 2021)	BLAST Top Hit Species	*E. coli*	*S. cerevisiae*
1	OK662633	M1_08	*Yokenella regensburgei*	−	−
2	OK662634	P2_02	*Pseudomonas koreensis*	−	−
3	OK662635	P2_06	*Pseudomonas vancouverensis*	−	−
4	OK662636	P2_15	*Serratia grimesii*	−	−
5	OK662637	P2_16	*Pseudomonas vancouverensis*	−	−
6	OK662638	P2_25	*Pseudomonas vancouverensis*	−	−
7	OK662639	P2_28	*Microbacterium hominis*	−	−
8	OK662640	P3_01	*Serratia grimesii*	−	−
9	OK662641	P3_07	*Serratia grimesii*	−	−
10	OK662959	P3_08	*Pseudomonas koreensis*	−	−
11	OK662642	P3_13	*Serratia grimesii*	−	−
12	OK662643	P3_17	*Serratia grimesii*	−	−
13	OK662644	P3_18	*Pseudomonas koreensis*	−	−
14	OK662645	P3_19	*Pseudomonas koreensis*	−	−
15	OK662646	P3_24	*Pseudomonas koreensis*	−	−
16	OK662647	P3_25	*Serratia grimesii strain*	−	−
17	OK662648	P3_26	*Pseudomonas koreensis*	−	−
18	OK662649	P3_27	*Pseudomonas koreensis*	−	−
19	OK662650	P3_28	*Serratia grimesii*	−	−
20	OK662651	P3_29	*Serratia grimesii*	−	−
21	OK662652	P3_30	*Serratia grimesii*	−	−
22	OK662653	R22_05	*Bacillus subtilis*	+/−	−
23	OK662654	R22_06	*Bacillus subtilis*	+/−	−
24	OK662655	R22_08	*Pseudomonas nitroreducens*	−	−
25	OK662656	R23_08	*Bacillus tequilensis*	−	−
26	OK662657	R23_12	*Bacillus subtilis*	+	−
27	OK662658	R23_17	*Bacillus cereus*	−	−
28	OK662659	R23_28	*Bacillus subtilis*	+++	−
29	OK662660	S1_29	*Enterobacter cloacae subsp. dissolvens*	+++	−
30	OK662661	S2_11	*Janthinobacterium lividum*	−	−
31	OK662662	S2_18	*Janthinobacterium lividum*	−	−

*1 Growth inhibition activities are shown in 5 classes from *“*−*”* to *“*+++*”*. +++, all the dilution series have more than 10% inhibition; +, only one dilution has more than 10% inhibition; +/−, only one dilution has more than 0% but less than 10% inhibition; −, no dilution series have more than 10% inhibition.

**Table 2:** Identification of lipopeptide signals present in the analysis by MALDI-TOF of the supernatant of samples of *B. subtilis* (strain 22, 23 26, 28) and *B. tequilensis* (strain 25) after 48 or 72 h cultivation.

*m*/*z*		Strain 22		Strain 23		Strain 25		Strain 26		Strain 28	
	Identification	48 h	72 h	48 h	72 h	48 h	72 h	48 h	72 h	48 h	72 h
724.44	Plipastatin B C13 [M + H_2_]^2+^ Plipastatin A C15 [M + H_2_]^2+^	+	+	+	+	+	+	+	+	+	+
746.43	Plipastatin B C13 [M + Na_2_]^2+^ Plipastatin A C15 [M + Na_2_]^2+^	+	+	+	+	+	+	+	+	+	+
762.42	Plipastatin B C13 [M + K_2_]^2+^ Plipastatin A C15 [M + K_2_]^2+^	+	+	+	+	+	+	+	+	+	+
781.35	Plipastatin B C18 [M + Na_2_]^2+^	-	-	+	-	-	+	-	-	-	-
1030.72	Surfactin C13 [M + Na]^+^ [Val7] Surfactin C14 [M + Na]^+^ [Ala4] Surfactin C15 [M + Na]^+^	+	+	+	+	-	-	+	+	+	+
1044.74	Surfactin C14 [M + Na]^+^ [Val7] Surfactin C15 [M + Na]^+^	+	+	+	+	-	+	+	+	+	+
1058.75	Surfactin C15 [M + Na]^+^ [Val7] Surfactin C16 [M + Na]^+^	+	+	+	+	-	+	+	+	+	+
1060.71	Surfactin C14 [M + K]^+^ [Val7] Surfactin C15 [M + K]^+^	+	+	+	+	-	+	+	+	+	+
1074.74	Surfactin C15 [M + K]^+^ [Val7] Surfactin C16 [M + K]^+^	+	+	+	+	-	+	+	+	+	+
1088.73	Surfactin C16 [M + K]^+^ [Val7] Surfactin C17 [M + K]^+^	+	+	+	+	-	-	+	+	+	+
1485.86	Plipastatin B C14 [M + Na]^+^ Plipastatin A C16 [M + Na]+	-	+	-	-	-	-	-	+	-	+
1515.89	Plipastatin B C15 [M + K]^+^ Plipastatin A C17 [M + K]^+^	+	+	+	+	-	-	-	-	+	+
1571.63	Plipastatin B C19 [M + K]^+^	-	-	+	-	+	-	+	+	+	-

**Table 3:** Predicted gene clusters of NRP and other secondary metabolites by antiSMASH in the genomes of *B. subtilis* strains (number 22, 23 26, and 28). The percentage refers to the similarity of a gene cluster to a reference gene cluster.

		*B. subtilis* Strains							
Cluster	Category	22	23	26	28	Remarks			
Bacilysin	Other	100%	100%	100%	100%				
Subtilosin A	Thiopeptide	100%	100%	100%	100%				
Bacillibactin	NRP	100%	100%	100%	100%				
Bacillaene	Polyketide + NRP	100%	100%	100%	100%				
Plipastatin	NRP	100%	80% 46%, 23%	100%	100%	possible peptide chain: Glu-D-Orn-Tyr-D-Thr-Glu-D-Val-Pro-Glu-D-Tyr-Ile			
Surfactin	NRP	82%	82%	82%	43%, 43%, 8%	possible peptide chain: Glu-Leu-D-Leu-Val-Asp-D-Leu-Leu			
Icosalide A/B	NRP				100%				
Sporulation killing factor	RiPP: Head-to-tail cyclized peptide	100%							

## Data Availability

Not applicable.

## Supplementary Materials

The following supporting information can be downloaded at: https://www.mdpi.com/article/10.3390/microorganisms10020209/s1, Figure S1: The layout of 96-well plates for growth inhibition test against E. coli and S. cerevisiae; Figure S2: Dual culture test of vetiver endophytes and F. culmorum (a) and F. oxysporum (b); Figure S3: Growth inhibition rate of the cell-free supernatants of vetiver endophytic bacterial strains in NB against F. graminearum, F. culmorum, and F. oxysporum; Figure S4: Growth inhibition rate of the cell-free supernatant of vetiver endophytic strains against E.coli (a) and S. cerevisiae (b); Figure S5: Growth inhibition rate of the solutions of commercial surfactins, plipastatins, and mixtures of the two lipopeptides against F. graminearum, F. culmorum, and F. oxysporum; Figure S6: Liquid handler protocol for the high-throughput screening assays; Figure S7: Liquid handler protocols for the MALDI-TOF target preparation; Table S1: Summary of the in vitro growth inhibition activities of the 31 vetiver endophytes against Fusarium plant pathogens with the bacterial cells or the cell-free supernatants.
