# Supplementary material for: Screening of Antimicrobial Activities and Lipopeptide Production of Endophytic Bacteria Isolated from Vetiver Roots
Source: Microorganisms. 2022 Jan 19;10(2):209. doi: 10.3390/microorganisms10020209 (PMC8876289; doi:10.3390/microorganisms10020209)
Supplement: Supplementary file 1 [file microorganisms-10-00209-s001.zip › microorganisms 1536927-supplementary materials.pdf]

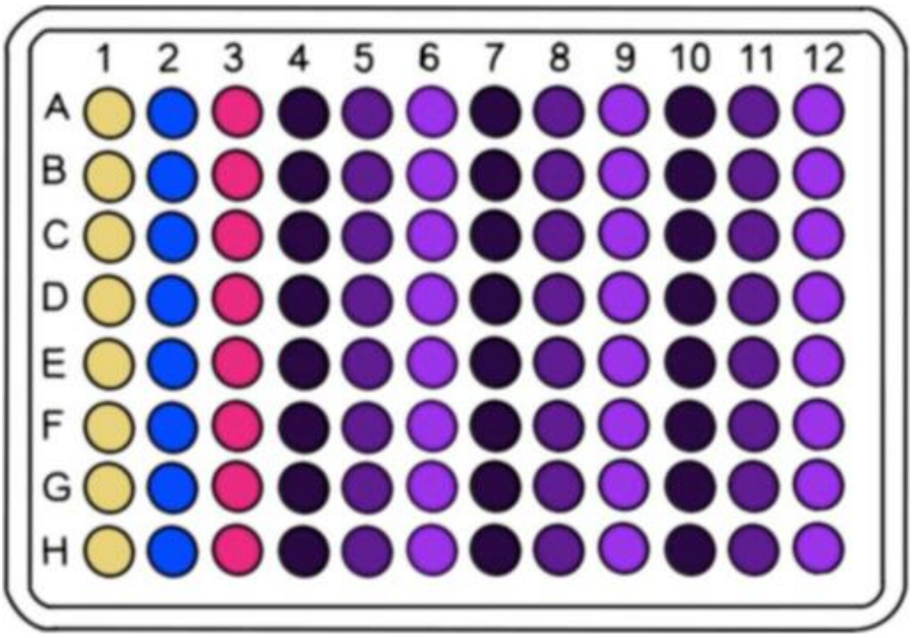

Figure S1 The layout of 96 well plates for growth inhibition test against *E. coli* and *S. cerevisiae*

Plate design for liquid antimicrobial activity testing. Each plate line is used to test a supernatant. Column 1(●) is the sterility control (200 µL of culture medium (Muller Hinton for *E. coli*, and YPG for *S. cerevisiae*)), column 2 (●) is the supernatant sterility control (50 µL of supernatant + 150 µL of culture medium), column 3 (●) corresponds to the growth control (100 µL of culture medium + 100 µL of target strain). Columns 4, 7 and 10 (●) correspond to growth in the culture medium in the presence of the tested supernatant diluted by 1/2 (100 µL of supernatant + 100 µL of inoculum). Columns 5, 8 and 11 (●) correspond to growth in culture medium in the presence of 1/4 diluted test supernatant (100 µL of mixture from columns 4, 7 and 10 respectively + 100 µL of culture medium – 100µL of mixture + 100 µL of inoculum). Columns 6, 9 and 12 (●) correspond to growth in culture medium in the presence of the 1/8 diluted test supernatant (100 µL of mixture from columns 5, 8 and 10 respectively + 100 µL of culture medium - 100 µL of mixture + 100 µL of inoculum).

Table S1 Summary of the growth inhibition rate in the dual-culture test and the diffusion assay of the cell-free supernatant samples against *F. graminearum*, *F. culmorum*, and *F. oxysporum*.

				Dual-culture test [*1]			Diffusion assay with cell-free supernatant [*1]					
							F. graminearum		*F. culmorum*		*F. oxysporum*	
Strain	Genbank accession	Accession (Munakata et al., 2021)	BLAST top hit species	*F. graminearum* (Munakata et al., 2021)	*F. culmorum*	*F. oxysporum*	King's B	NB	King's B	NB	King's B	NB
1	OK662633	M1_08	*Yokenella regensburgei*	71.3	26.8 ± 5	27.7 ± 8.1	0 ± 0	1.9 ± 2.6	2.3 ± 3.3	11 ± 13.5	0 ± 0	1.0 ± 1.3
2	OK662634	P2_02	*Pseudomonas koreensis*	73.5	11.4 ± 12.1	1.7 ± 1.9	0 ± 0	1.9 ± 2.6	0 ± 0	2.2 ± 3.1	0 ± 0	0.9 ± 1.3
3	OK662635	P2_06	*Pseudomonas vancouverensis*	78.5	8.8 ± 8.4	18.9 ± 17.4	2.0 ± 2.9	3.0 ± 2.6	0 ± 0	5.3 ± 3.3	0 ± 0	0 ± 0
4	OK662636	P2_15	*Serratia grimesii*	73.1	46.2 ± 3.3	48.7 ± 7.7	5.1 ± 7.1	2.7 ± 3.8	0 ± 0	6.0 ± 4.2	17.7 ± 14.1	1.8 ± 2.5
5	OK662637	P2_16	*Pseudomonas vancouverensis*	72.1	1.1 ± 1.9	17.4 ± 3.5	0 ± 0	0.9 ± 1.3	0 ± 0	2.0 ± 2.8	8.0 ± 9.2	4.4 ± 2.3
6	OK662638	P2_25	*Pseudomonas vancouverensis*	79.2	3.2 ± 5.6	6.1 ± 10.5	0 ± 0	4.8 ± 2.5	4.0 ± 2.8	0 ± 0	6.9 ± 9.8	4.3 ± 3.2
7	OK662639	P2_28	*Microbacterium hominis*	70.6	10.9 ± 6.2	0 ± 0	4.2 ± 5.9	4.2 ± 5.9	4.8 ± 6.7	1.0 ± 1.5	0 ± 0	0.9 ± 1.2
8	OK662640	P3_01	*Serratia grimesii*	72.1	45.3 ± 2.7	48.1 ± 9.6	5.2 ± 5.3	5.9 ± 4.8	4.4 ± 4.4	2.9 ± 4.0	2.1 ± 2.9	0 ± 0
9	OK662641	P3_07	*Serratia grimesii*	73.1	46.1 ± 4	37.2 ± 27.5	2.0 ± 1.4	5.8 ± 5.0	4.4 ± 4.4	4.1 ± 3.5	2.9 ± 4.0	3.5 ± 3.3
10	OK662959	P3_08	*Pseudomonas koreensis*	71.0	16.5 ± 11.7	0.2 ± 0.4	4.9 ± 4.8	4.4 ± 6.2	1.0 ± 1.4	4.2 ± 3.0	1.8 ± 2.5	0.9 ± 1.3
11	OK662642	P3_13	*Serratia grimesii*	74.9	45.7 ± 4.6	48.3 ± 4.2	3.1 ± 2.3	4.5 ± 1.2	0 ± 0	5.7 ± 5.9	6.2 ± 6.9	1.8 ± 2.5
12	OK662643	P3_17	*Serratia grimesii*	73.8	39.9 ± 5.2	46.6 ± 3	1.9 ± 2.6	3.7 ± 2.6	1.0 ± 1.4	4.6 ± 3.3	0 ± 0	0.9 ± 1.2
13	OK662644	P3_18	*Pseudomonas koreensis*	70.6	8.6 ± 11.9	6.7 ± 2.4	2.8 ± 3.9	1.9 ± 1.4	0 ± 0	6.2 ± 4.5	0.9 ± 1.2	0.9 ± 1.2
14	OK662645	P3_19	*Pseudomonas koreensis*	71.7	12.4 ± 6.7	11.6 ± 1.5	2.9 ± 2.3	3.1 ± 4.4	1.0 ± 1.5	5.6 ± 6.0	0 ± 0	0.9 ± 1.3
15	OK662646	P3_24	*Pseudomonas koreensis*	74.2	15.3 ± 5.6	1.9 ± 3.3	3.2 ± 2.6	1.1 ± 1.5	4.3 ± 1.8	1.9 ± 2.6	0 ± 0	2.7 ± 2.2
16	OK662647	P3_25	*Serratia grimesii strain*	73.1	43.1 ± 5.1	47 ± 6.3	6.3 ± 6.8	2.0 ± 2.8	6.1 ± 2.2	0 ± 0	3.8 ± 3.4	0 ± 0
17	OK662648	P3_26	*Pseudomonas koreensis*	72.1	23.9 ± 6.8	4.8 ± 4.3	2.1 ± 2.9	10 ± 9.9	2.0 ± 2.9	1.9 ± 2.7	0 ± 0	0 ± 0
18	OK662649	P3_27	*Pseudomonas koreensis*	73.8	11.2 ± 10	4.6 ± 7.9	1.1 ± 1.6	4.9 ± 6.9	1.0 ± 1.4	1.9 ± 2.7	1.9 ± 2.7	0 ± 0
19	OK662650	P3_28	*Serratia grimesii*	78.1	49.9 ± 5.2	46 ± 3.1	2.2 ± 3.1	1.0 ± 1.4	0 ± 0	1.9 ± 1.4	1.9 ± 2.7	0 ± 0
20	OK662651	P3_29	*Serratia grimesii*	79.9	53.4 ± 7	55.3 ± 9.6	3.2 ± 2.6	7.9 ± 6.2	2.0 ± 2.8	1.0 ± 1.4	0 ± 0	0 ± 0
21	OK662652	P3_30	*Serratia grimesii*	73.5	51.2 ± 3.5	55.2 ± 5.8	3.2 ± 2.6	5.0 ± 5.2	0 ± 0	1.0 ± 1.4	1.0 ± 1.3	1.0 ± 1.3
22	OK662653	R22_05	*Bacillus subtilis*	81.7	45.8 ± 2.9	33 ± 5.6	35.4 ± 0.8	38.1 ± 4.9	36.8 ± 1.0	24.3 ± 3.8	31.9 ± 14.7	0 ± 0
23	OK662654	R22_06	*Bacillus subtilis*	81.7	46.5 ± 5.2	39.2 ± 2.8	37.8 ± 1.2	39.7 ± 4.5	32.5 ± 6.1	22.6 ± 17.6	42.8 ± 1.3	0 ± 0
24	OK662655	R22_08	*Pseudomonas nitroreducens*	70.0	26.4 ± 9.7	27.7 ± 6.5	4.1 ± 3.8	4.0 ± 3.8	1.1 ± 1.6	1.0 ± 1.3	1.9 ± 2.6	0 ± 0
25	OK662656	R23_08	*Bacillus tequilensis*	75.0	37.7 ± 7.9	41 ± 0.2	3.1 ± 2.5	4.1 ± 1.4	3.4 ± 4.9	1.9 ± 2.7	0 ± 0	0.9 ± 1.3
26	OK662657	R23_12	*Bacillus subtilis*	70.0	47.4 ± 2.2	41.1 ± 4.3	37.5 ± 1.6	34.1 ± 5.9	32.2 ± 4.8	31.1 ± 4.7	28.6 ± 20.2	31.5 ± 14.8
27	OK662658	R23_17	*Bacillus cereus*	78.3	19.6 ± 3.5	36.2 ± 4	3.2 ± 2.6	6.9 ± 3.9	2.0 ± 2.9	0 ± 0	0 ± 0	0.9 ± 1.3
28	OK662659	R23_28	*Bacillus subtilis*	73.3	47 ± 2.8	40.5 ± 4	35.4 ± 1.7	42.9 ± 0	34.3 ± 1.4	38.8 ± 4.0	26.2 ± 18.6	42.9 ± 2.4
29	OK662660	S1_29	*Enterobacter cloacae subsp. dissolvens*	82.8	47.4 ± 10	N.D.	3.3 ± 4.7	4.9 ± 2.6	2.2 ± 3.1	3.7 ± 3.5	1.0 ± 1.3	0 ± 0
30	OK662661	S2_11	*Janthinobacterium lividum*	82.5	7.3 ± 6.8	30 ± 15.8	0 ± 0	4.1 ± 3.9	0 ± 0	2.9 ± 4.0	16.2 ± 19.0	2.8 ± 3.9
31	OK662662	S2_18	*Janthinobacterium lividum*	76.0	34 ± 11	0 ± 0	1.1 ± 1.5	0.9 ± 1.3	0 ± 0	1.0 ± 1.3	0 ± 0	1.9 ± 2.6

*1 Growth inhibition rate is shown as mean value ± SD. More than 30% growth inhibition is shown in gray (n = 3)

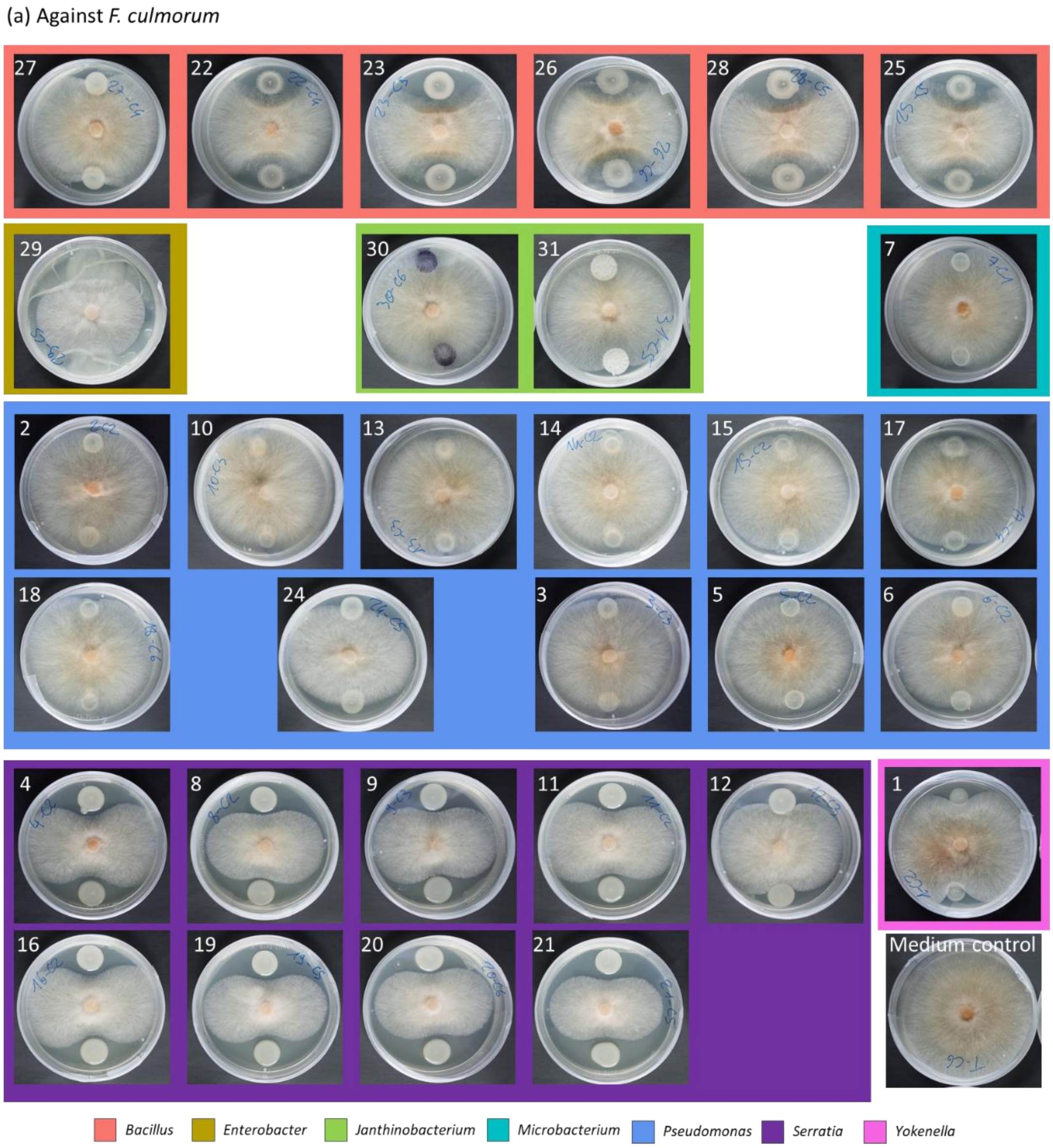

Figure S2 Dual culture test of Vetiver endophytes against *F. culmorum* (a) and *F. oxysporum* (b)

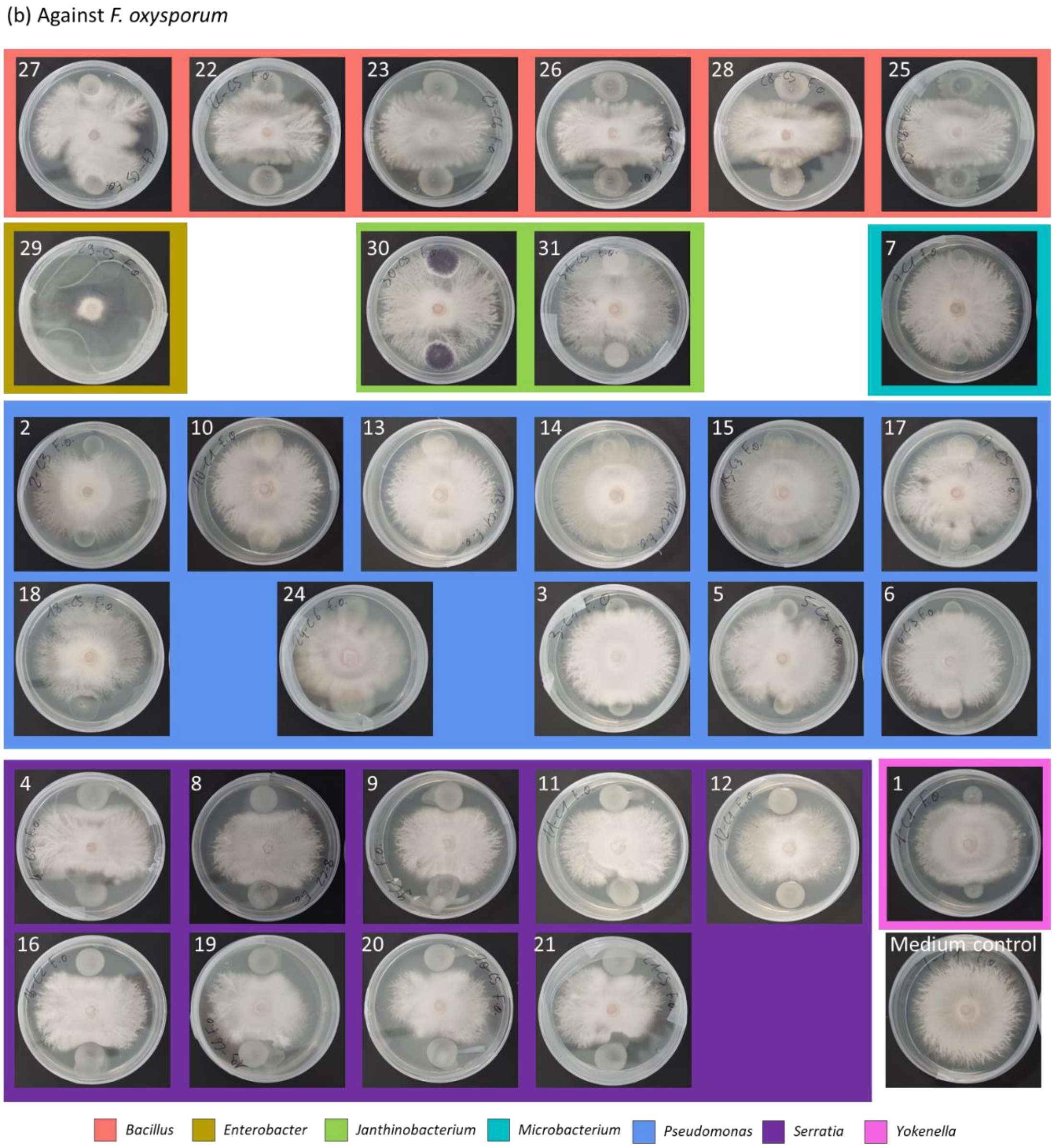

Figure S2 (Continued)

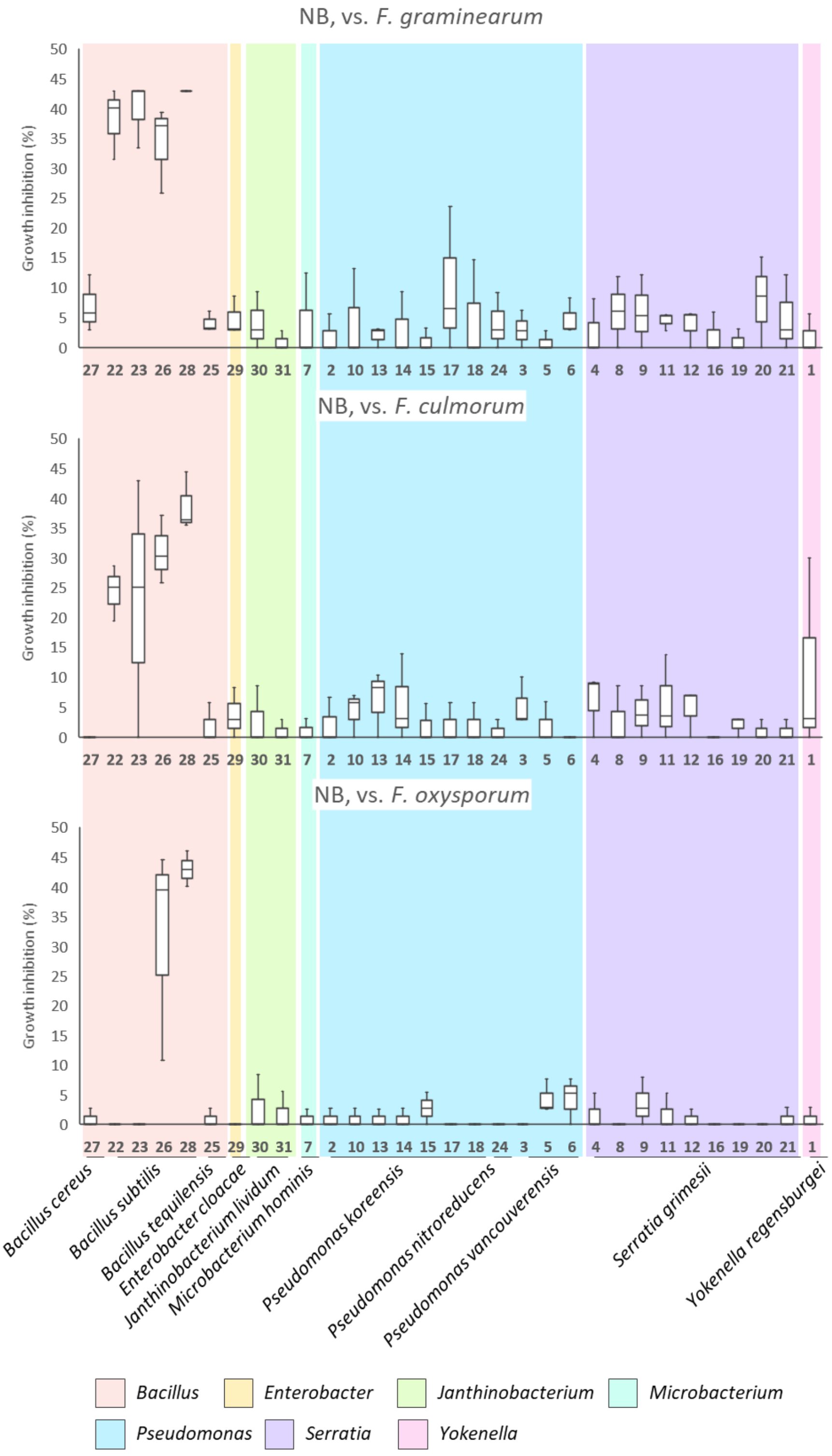

Figure S3 Growth inhibition rate of the cell-free supernatants of vetiver endophytic bacterial strains in NB against *F. graminearum*, *F. culmorum*, and *F. oxysporum* (n = 3)

(a) *E. coli*

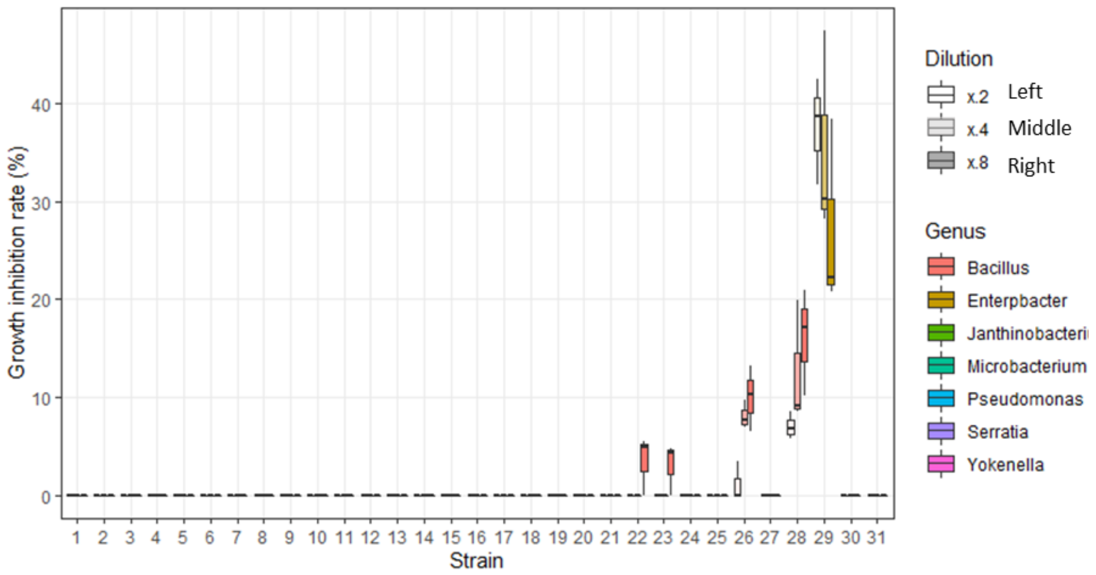

(b) *S. cerevisiae*

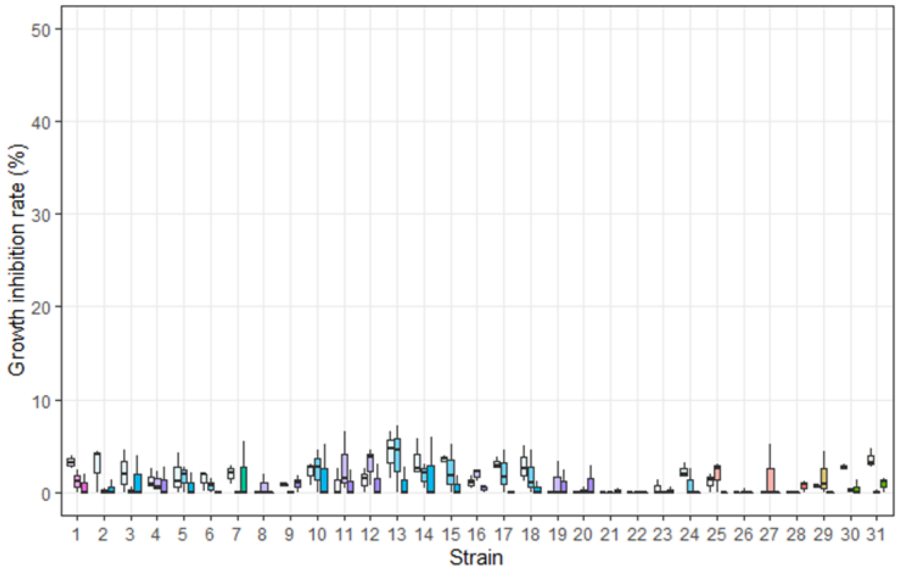

Figure S4 Growth inhibition rate of the cell-free supernatant of Vetiver endophytic strains against *E. coli* (a) and *S. cerevisiae* (b) (n = 3)

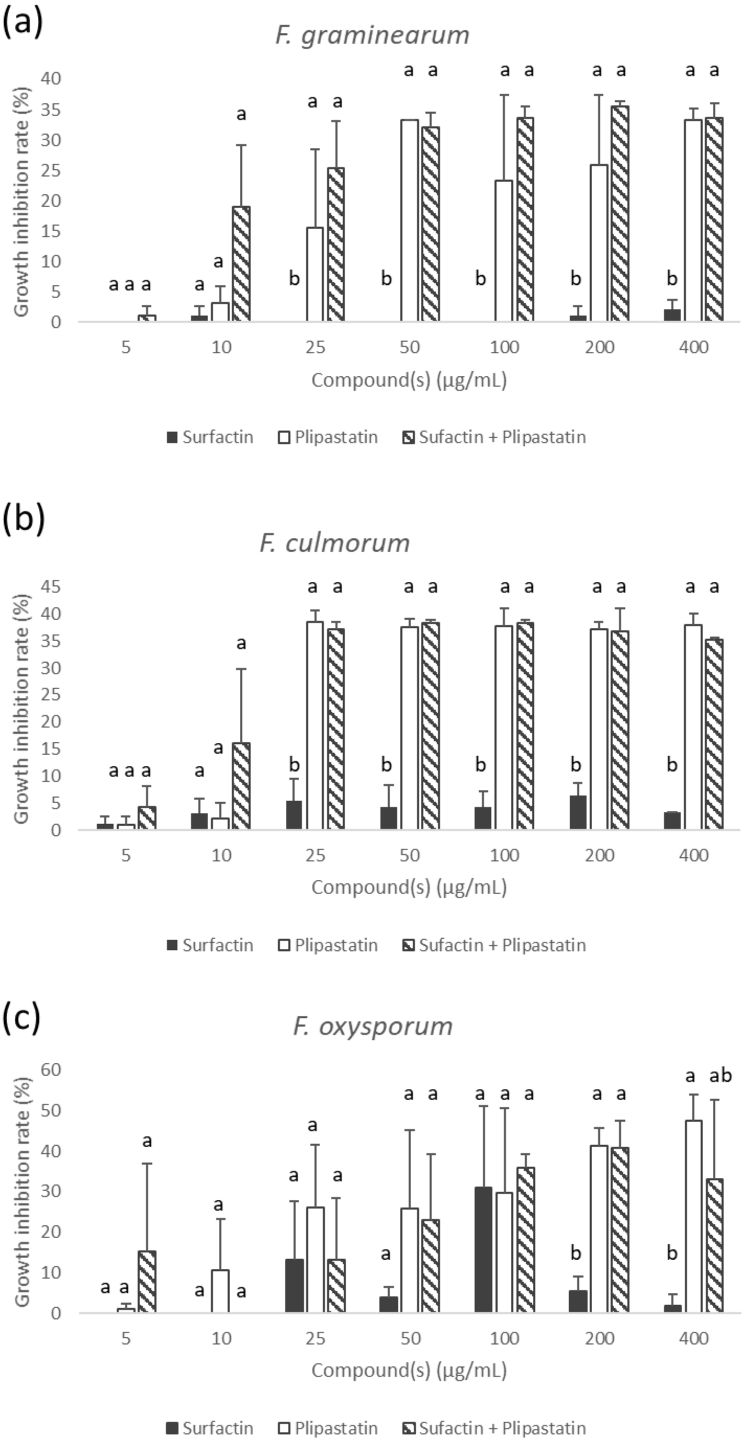

Figure S5 Growth inhibition rate of the solutions of commercial surfactins, plipastatins, and mixtures of the two lipopeptides against *F. graminearum* (a), *F. culmorum* (b), and *F. oxysporum*.(c). For each concentration, the same alphabets have no significant difference within a concentration (Tukey-Kramer test, $p < 0.05$). (Error bars = standard deviation, n = 3)

Figure S6 Liquid handler protocol for the high-throughput screening assays

REALCAT\Antimicrobial Activities\AntiMAct_Liquid_CMI_RTU_v2

12/3/2021 3:49:10 PM Page:1

Method

Author: Beckman Coulter Inc

Description:

Start

Define the following values for this method:

targStrainVol = 30000

targStrainNum = 3

speedPercent = 100

nbrOfMix = 5

nbrOfBatch = 3

firstColNum = 1

brothVol = 30000

brothNum = 1

Comment

Description:

Initial Setup

Comment:

Variables:

"brothNum": Stores the number of broth reservoir to use in the run (1 - 2)

"targStrainNum": Stores the number of inoculum reservoir to use in the run (3 - 4)

"firstColNum": Stores the column number of the first batch of supernatants to use (1 - 12)

"nbrOfBatch": Stores the number of batch (columns of supernatants & target plates) to use

and create during the run (1 - 3)

"nbrOfMix": Stores the number of mix action to perform at each mixing step

"speedPercent": Stores the move speed in wells for the tips

Plates:

"Batch_1": Empty 96_Plate for Vis reading at 595 nm

"Batch_2": Empty 96_Plate for Vis reading at 595 nm

"Batch_3": Empty 96_Plate for Vis reading at 595 nm

"Supernatants" : min 800µL of filtrated supernatant by well sorted in column (Batch)

/!\The batchs must be placed every two columns for the same run!

(Each batch corresponds to column of supernatants in the "Supernatants" plate - The

analysis are made in technical triplicates in each plate (Batch)))

Reservoirs:

"Broth1 + Broth2 + Inoc1 + Inoc2": 30000µL broth1 + 30000µL broth2 + 30000µL Inoculum

Strain 1 (OD = 0.1) + 30000µL Inoculum Strain 2 (OD = 0.1)

(Only one broth and inoculum is used per run of program. The two slots are here to have

the opportunity to choose between two targets and broths)

"Trash": empty reservoir

Instrument Setup

Deck: REALCAT_BKL

Pause to confirm setup.

Verify that the pod is set up in its default configuration.

Items:

FM1: Nothing

Holder1: Nothing

IN1: Nothing

MID1: Nothing

OUT1: Nothing

P1: Nothing

P10: Reserv_Full_Beckman named Trash with known volume: 0 µL of Water in all wells

Print Error Instrument Setup

Loop

Loop from "a" = "1" to "=nbrOfBatch", incrementing by "1".

Move Labware

Move the top "1" plates at "="P"&a+3" to "="P"&a" using pod "Pod1".

Group:

Broth Filling

Span-8 New Tips

Get new P200B tips for all probes on Pod2.

Group:

Col1

Loop

Loop from "1" to "2", incrementing by "1".

Span-8 Aspirate

Using Pod2, Aspirate 100 µL from the labware at Broth1 + Broth2 + Inoc1 + Inoc2 using the

EHw_Span-8t Reserv technique.

All probes will be used, with a spacing of 1.

The first selected probe will pipette from well =brothNum.

Override the technique height by moving to -5 mm from the liquid.

The liquid type is Water and the expected labware type is Reserv_Modular_40mL.

Span-8 Dispense

Using Pod2, Dispense 100 µL to the labware at ="Batch_"&a using the EHw_Span-8t technique.

All probes will be used, with a spacing of 1.

The first selected probe will pipette to well 1.

Override the technique height by moving to 0 mm from the liquid.

The liquid type is Water and the expected labware type is Plate_96_F_Vis_Greiner.

End Loop

End Group

Group:

Col2

Loop

Loop from "1" to "2", incrementing by "1".

Span-8 Aspirate

Using Pod2, Aspirate 75 µL from the labware at Broth1 + Broth2 + Inoc1 + Inoc2 using the

EHw_Span-8t Reserv technique.

All probes will be used, with a spacing of 1.

The first selected probe will pipette from well =brothNum.

Override the technique height by moving to -5 mm from the liquid.

The liquid type is Water and the expected labware type is Reserv_Modular_40mL.

Span-8 Dispense

Using Pod2, Dispense 75 µL to the labware at ="Batch_"&a using the EHw_Span-8t technique.

All probes will be used, with a spacing of 1.

The first selected probe will pipette to well 2.

Override the technique height by moving to 0 mm from the liquid.

The liquid type is Water and the expected labware type is Plate_96_F_Vis_Greiner.

End Loop

End Group

Group:

Col3

Span-8 Aspirate

Using Pod2, Aspirate 100 µL from the labware at Broth1 + Broth2 + Inoc1 + Inoc2 using the

EHw_Span-8t Reserv technique.

All probes will be used, with a spacing of 1.

The first selected probe will pipette from well =brothNum.

Override the technique height by moving to -5 mm from the liquid.

The liquid type is Water and the expected labware type is Reserv_Modular_40mL.

Span-8 Dispense

Using Pod2, Dispense 100 µL to the labware at ="Batch_"&a using the EHw_Span-8t technique.

All probes will be used, with a spacing of 1.

The first selected probe will pipette to well 3.

Override the technique height by moving to 0 mm from the liquid.

The liquid type is Water and the expected labware type is Plate_96_F_Vis_Greiner.

End Group

Group:

Col5,6,8,9,11,12

Loop

Loop from "b" = "1" to "7", incrementing by "3".

Loop

Loop from "c" = "0" to "1", incrementing by "1".

Span-8 Aspirate

Using Pod2, Aspirate 100 µL from the labware at Broth1 + Broth2 + Inoc1 + Inoc2 using the

EHw_Span-8t Reserv technique.

All probes will be used, with a spacing of 1.

The first selected probe will pipette from well =brothNum.

Override the technique height by moving to -5 mm from the liquid.

The liquid type is Water and the expected labware type is Reserv_Modular_40mL.

Span-8 Dispense

Using Pod2, Dispense 100 µL to the labware at ="Batch_"&a using the EHw_Span-8t technique.

All probes will be used, with a spacing of 1.

The first selected probe will pipette to well =4+b+c.

Override the technique height by moving to 0 mm from the liquid.

The liquid type is Water and the expected labware type is Plate_96_F_Vis_Greiner.

End Loop

End Loop

End Group

End Group

Group:

Supernatent Filling

Span-8 New Tips

Get new P200B tips for all probes on Pod2.

Move Labware

Move the top "1" plates at "P8" to "P9" using pod "Pod1".

Group:

Col2

If

If "=a=1":

Then

Span-8 Aspirate

Using Pod2, Aspirate 50 µL from the labware at Supernatents using the EHw_Span-8t

technique.

All probes will be used, with a spacing of 1.

The first selected probe will pipette from well =firstColNum.

Override the technique height by moving to -2 mm from the liquid.

The liquid type is Water and the expected labware type is DeepWell_96_U_Square_Greiner.

Span-8 Dispense

Using Pod2, Dispense 50 µL to the labware at ="Batch_"&a using the EHw_Span-8t technique.

All probes will be used, with a spacing of 1.

The first selected probe will pipette to well 2.

Override the technique height by moving to 0 mm from the liquid.

The liquid type is Water and the expected labware type is Plate_96_F_Vis_Greiner.

End

Else

If

If "=a=2":

Then

Span-8 Aspirate

Using Pod2, Aspirate 50 µL from the labware at Supernatents using the EHw_Span-8t

technique.

All probes will be used, with a spacing of 1.

The first selected probe will pipette from well =firstColNum+2.

Override the technique height by moving to -2 mm from the liquid.

The liquid type is Water and the expected labware type is DeepWell_96_U_Square_Greiner.

Span-8 Dispense

Using Pod2, Dispense 50 µL to the labware at ="Batch_"&a using the EHw_Span-8t technique.

All probes will be used, with a spacing of 1.

The first selected probe will pipette to well 2.

Override the technique height by moving to 0 mm from the liquid.

The liquid type is Water and the expected labware type is Plate_96_F_Vis_Greiner.

End

Else

Span-8 Aspirate

Using Pod2, Aspirate 50 µL from the labware at Supernatents using the EHw_Span-8t

technique.

All probes will be used, with a spacing of 1.

The first selected probe will pipette from well =firstColNum+4.

Override the technique height by moving to -2 mm from the liquid.

The liquid type is Water and the expected labware type is DeepWell_96_U_Square_Greiner.

Span-8 Dispense

Using Pod2, Dispense 50 µL to the labware at ="Batch_"&a using the EHw_Span-8t technique.

All probes will be used, with a spacing of 1.

The first selected probe will pipette to well 2.

Override the technique height by moving to 0 mm from the liquid.

The liquid type is Water and the expected labware type is Plate_96_F_Vis_Greiner.

End

End

End Group

Group:

Col4,7,10

If

If "=a=1":

Then

Loop

Loop from "d" = "4" to "10", incrementing by "3".

Span-8 Aspirate

Using Pod2, Aspirate 100 µL from the labware at Supernatents using the EHw_Span-8t

technique.

All probes will be used, with a spacing of 1.

The first selected probe will pipette from well =firstColNum.

Override the technique height by moving to -2 mm from the liquid.

The liquid type is Water and the expected labware type is DeepWell_96_U_Square_Greiner.

Span-8 Dispense

Using Pod2, Dispense 100 µL to the labware at ="Batch_"&a using the EHw_Span-8t technique.

All probes will be used, with a spacing of 1.

The first selected probe will pipette to well =d.

Override the technique height by moving to 0 mm from the liquid.

The liquid type is Water and the expected labware type is Plate_96_F_Vis_Greiner.

End Loop

End

Else

If

If "=a=2":

Then

Loop

Loop from "d" = "4" to "10", incrementing by "3".

Span-8 Aspirate

Using Pod2, Aspirate 100 µL from the labware at Supernatents using the EHw_Span-8t

technique.

All probes will be used, with a spacing of 1.

The first selected probe will pipette from well =firstColNum+2.

Override the technique height by moving to -2 mm from the liquid.

The liquid type is Water and the expected labware type is DeepWell_96_U_Square_Greiner.

Span-8 Dispense

Using Pod2, Dispense 100 µL to the labware at ="Batch_"&a using the EHw_Span-8t technique.

All probes will be used, with a spacing of 1.

The first selected probe will pipette to well =d.

Override the technique height by moving to 0 mm from the liquid.

The liquid type is Water and the expected labware type is Plate_96_F_Vis_Greiner.

End Loop

End

Else

Loop

Loop from "d" = "4" to "10", incrementing by "3".

Span-8 Aspirate

Using Pod2, Aspirate 100 µL from the labware at Supernatents using the EHw_Span-8t

technique.

All probes will be used, with a spacing of 1.

The first selected probe will pipette from well =firstColNum+4.

Override the technique height by moving to -2 mm from the liquid.

The liquid type is Water and the expected labware type is DeepWell_96_U_Square_Greiner.

Span-8 Dispense

Using Pod2, Dispense 100 µL to the labware at ="Batch_"&a using the EHw_Span-8t technique.

All probes will be used, with a spacing of 1.

The first selected probe will pipette to well =d.

Override the technique height by moving to 0 mm from the liquid.

The liquid type is Water and the expected labware type is Plate_96_F_Vis_Greiner.

End Loop

End

End

End Group

Group:

Col5,8,11

If

If "=a=1":

Then

Loop

Loop from "d" = "5" to "11", incrementing by "3".

Span-8 Aspirate

Using Pod2, Aspirate 100 µL from the labware at Supernatents using the EHw_Span-8t

technique.

All probes will be used, with a spacing of 1.

The first selected probe will pipette from well =firstColNum.

Override the technique height by moving to -2 mm from the liquid.

The liquid type is Water and the expected labware type is DeepWell_96_U_Square_Greiner.

Span-8 Dispense

Using Pod2, Dispense 100 µL to the labware at ="Batch_"&a using the following technique:

Use the following pipetting template: Span-8

Calibration Offset: 0

Calibration Slope: 1.05

Minimum Pipetting Height: 0.5 mm

Prewet: True

Blowout: True

Follow Liquid: True

Height: 1.5 mm from the liquid

Mix: True

Mix Aspirate Speed: 100µL/s

Mix Aspirate Height: 1.5 mm from the bottom

Mix Dispense Speed: 400µL/s

Mix Dispense Height: -1.5 mm from the liquid

Mix Count: =nbrOfMix

Mix Volume: 50 µL

Operation speed: 5µL/s

Tip Touch: False

All probes will be used, with a spacing of 1.

The first selected probe will pipette to well =d.

Override the technique height by moving to 0 mm from the liquid.

The liquid type is Water and the expected labware type is Plate_96_F_Vis_Greiner.

End Loop

End

Else

If

If "=a=2":

Then

Loop

Loop from "d" = "5" to "11", incrementing by "3".

Span-8 Aspirate

Using Pod2, Aspirate 100 µL from the labware at Supernatents using the EHw_Span-8t

technique.

All probes will be used, with a spacing of 1.

The first selected probe will pipette from well =firstColNum+2.

Override the technique height by moving to -2 mm from the liquid.

The liquid type is Water and the expected labware type is DeepWell_96_U_Square_Greiner.

Span-8 Dispense

Using Pod2, Dispense 100 µL to the labware at ="Batch_"&a using the following technique:

Use the following pipetting template: Span-8

Calibration Offset: 0

Calibration Slope: 1.05

Minimum Pipetting Height: 0.5 mm

Prewet: True

Blowout: True

Follow Liquid: True

Height: 1.5 mm from the liquid

Mix: True

Mix Aspirate Speed: 100µL/s

Mix Aspirate Height: 1.5 mm from the bottom

Mix Dispense Speed: 400µL/s

Mix Dispense Height: -1.5 mm from the liquid

Mix Count: =nbrOfMix

Mix Volume: 50 µL

Operation speed: 5µL/s

Tip Touch: False

All probes will be used, with a spacing of 1.

The first selected probe will pipette to well =d.

Override the technique height by moving to 0 mm from the liquid.

The liquid type is Water and the expected labware type is Plate_96_F_Vis_Greiner.

End Loop

End

Else

Loop

Loop from "d" = "5" to "11", incrementing by "3".

Span-8 Aspirate

Using Pod2, Aspirate 100 µL from the labware at Supernatents using the EHw_Span-8t

technique.

All probes will be used, with a spacing of 1.

The first selected probe will pipette from well =firstColNum+4.

Override the technique height by moving to -2 mm from the liquid.

The liquid type is Water and the expected labware type is DeepWell_96_U_Square_Greiner.

Span-8 Dispense

Using Pod2, Dispense 100 µL to the labware at ="Batch_"&a using the following technique:

Use the following pipetting template: Span-8

Calibration Offset: 0

Calibration Slope: 1.05

Minimum Pipetting Height: 0.5 mm

Prewet: True

Blowout: True

Follow Liquid: True

Height: 1.5 mm from the liquid

Mix: True

Mix Aspirate Speed: 100µL/s

Mix Aspirate Height: 1.5 mm from the bottom

Mix Dispense Speed: 400µL/s

Mix Dispense Height: -1.5 mm from the liquid

Mix Count: =nbrOfMix

Mix Volume: 50 µL

Operation speed: 5µL/s

Tip Touch: False

All probes will be used, with a spacing of 1.

The first selected probe will pipette to well =d.

Override the technique height by moving to 0 mm from the liquid.

The liquid type is Water and the expected labware type is Plate_96_F_Vis_Greiner.

End Loop

End

End

End Group

Move Labware

Move the top "1" plates at "P9" to "P8" using pod "Pod1".

Group:

Col6,9,12

Loop

Loop from "d" = "5" to "11", incrementing by "3".

Span-8 Aspirate

Using Pod2, Aspirate 100 µL from the labware at ="Batch_"&a using the following technique:

Use the following pipetting template: Span-8

Calibration Offset: 0

Calibration Slope: 1.05

Minimum Pipetting Height: 0.5 mm

Prewet: True

Aspirate Blowout: True

Follow Liquid: True

Height: -1.5 mm from the liquid

Mix: True

Mix Aspirate Speed: 100µL/s

Mix Aspirate Height: 1.5 mm from the liquid

Mix Dispense Speed: 400µL/s

Mix Dispense Height: -1.5 mm from the liquid

Mix Count: =nbrOfMix

Mix Volume: 50 µL

Operation speed: 5µL/s

Tip Touch: False

Trailing Air Gap: True

All probes will be used, with a spacing of 1.

The first selected probe will pipette from well =d.

Override the technique height by moving to -2 mm from the liquid.

The liquid type is Water and the expected labware type is Plate_96_F_Vis_Greiner.

Span-8 Dispense

Using Pod2, Dispense 100 µL to the labware at ="Batch_"&a using the following technique:

Use the following pipetting template: Span-8

Calibration Offset: 0

Calibration Slope: 1.05

Minimum Pipetting Height: 0.5 mm

Prewet: True

Blowout: True

Follow Liquid: True

Height: 1.5 mm from the liquid

Mix: True

Mix Aspirate Speed: 100µL/s

Mix Aspirate Height: 1.5 mm from the bottom

Mix Dispense Speed: 400µL/s

Mix Dispense Height: -1.5 mm from the liquid

Mix Count: =nbrOfMix

Mix Volume: 50 µL

Operation speed: 5µL/s

Tip Touch: False

All probes will be used, with a spacing of 1.

The first selected probe will pipette to well =d+1.

Override the technique height by moving to 0 mm from the liquid.

The liquid type is Water and the expected labware type is Plate_96_F_Vis_Greiner.

End Loop

Loop

Loop from "d" = "6" to "12", incrementing by "3".

Span-8 Aspirate

Using Pod2, Aspirate 100 µL from the labware at ="Batch_"&a using the EHw_Span-8t

technique.

All probes will be used, with a spacing of 1.

The first selected probe will pipette from well =d.

Override the technique height by moving to -2 mm from the liquid.

The liquid type is Water and the expected labware type is Plate_96_F_Vis_Greiner.

Span-8 Dispense

Using Pod2, Dispense 100 µL to the labware at Trash using the EHw_Span-8t Reserv

technique.

All probes will be used, with a spacing of 1.

The first selected probe will pipette to well 1.

Override the technique height by moving to 2 mm from the liquid.

The liquid type is Water and the expected labware type is Reserv_Full_Beckman.

End Loop

End Group

End Group

Group:

Innoculum Filling

Span-8 New Tips

Get new P200B tips for all probes on Pod2.

Group:

Col3

Span-8 Aspirate

Using Pod2, Aspirate 100 µL from the labware at Broth1 + Broth2 + Inoc1 + Inoc2 using the

EHw_Span-8t Reserv technique.

All probes will be used, with a spacing of 1.

The first selected probe will pipette from well =targStrainNum.

Override the technique height by moving to -4 mm from the liquid.

The liquid type is Water and the expected labware type is Reserv_Modular_40mL.

Span-8 Dispense

Using Pod2, Dispense 100 µL to the labware at ="Batch_"&a using the following technique:

Use the following pipetting template: Span-8

Calibration Offset: 0

Calibration Slope: 1.05

Minimum Pipetting Height: 0.5 mm

Prewet: True

Blowout: True

Follow Liquid: True

Height: 1.5 mm from the liquid

Mix: True

Mix Aspirate Speed: 100µL/s

Mix Aspirate Height: 1.5 mm from the bottom

Mix Dispense Speed: 400µL/s

Mix Dispense Height: -1.5 mm from the liquid

Mix Count: =nbrOfMix

Mix Volume: 50 µL

Operation speed: 5µL/s

Tip Touch: False

All probes will be used, with a spacing of 1.

The first selected probe will pipette to well 3.

Override the technique height by moving to 0 mm from the liquid.

The liquid type is Water and the expected labware type is Plate_96_F_Vis_Greiner.

End Group

Group:

Col6,9,12

Loop

Loop from "d" = "6" to "12", incrementing by "3".

Span-8 Aspirate

Using Pod2, Aspirate 100 µL from the labware at Broth1 + Broth2 + Inoc1 + Inoc2 using the

EHw_Span-8t Reserv technique.

All probes will be used, with a spacing of 1.

The first selected probe will pipette from well =targStrainNum.

Override the technique height by moving to -4 mm from the liquid.

The liquid type is Water and the expected labware type is Reserv_Modular_40mL.

Span-8 Dispense

Using Pod2, Dispense 100 µL to the labware at ="Batch_"&a using the EHw_Span-8t technique.

All probes will be used, with a spacing of 1.

The first selected probe will pipette to well =d.

Override the technique height by moving to 0 mm from the liquid.

The liquid type is Water and the expected labware type is Plate_96_F_Vis_Greiner.

End Loop

End Group

Group:

Col5,8,11

Loop

Loop from "d" = "5" to "11", incrementing by "3".

Span-8 Aspirate

Using Pod2, Aspirate 100 µL from the labware at Broth1 + Broth2 + Inoc1 + Inoc2 using the

EHw_Span-8t Reserv technique.

All probes will be used, with a spacing of 1.

The first selected probe will pipette from well =targStrainNum.

Override the technique height by moving to -4 mm from the liquid.

The liquid type is Water and the expected labware type is Reserv_Modular_40mL.

Span-8 Dispense

Using Pod2, Dispense 100 µL to the labware at ="Batch_"&a using the EHw_Span-8t technique.

All probes will be used, with a spacing of 1.

The first selected probe will pipette to well =d.

Override the technique height by moving to 0 mm from the liquid.

The liquid type is Water and the expected labware type is Plate_96_F_Vis_Greiner.

End Loop

End Group

Group:

Col5,8,11

Loop

Loop from "d" = "4" to "10", incrementing by "3".

Span-8 Aspirate

Using Pod2, Aspirate 100 µL from the labware at Broth1 + Broth2 + Inoc1 + Inoc2 using the

EHw_Span-8t Reserv technique.

All probes will be used, with a spacing of 1.

The first selected probe will pipette from well =targStrainNum.

Override the technique height by moving to -4 mm from the liquid.

The liquid type is Water and the expected labware type is Reserv_Modular_40mL.

Span-8 Dispense

Using Pod2, Dispense 100 µL to the labware at ="Batch_"&a using the EHw_Span-8t technique.

All probes will be used, with a spacing of 1.

The first selected probe will pipette to well =d.

Override the technique height by moving to 0 mm from the liquid.

The liquid type is Water and the expected labware type is Plate_96_F_Vis_Greiner.

End Loop

End Group

Group:

Mixings

Loop

Loop from "d" = "6" to "12", incrementing by "3".

Span-8 Aspirate

Using Pod2, Aspirate 0 µL from the labware at ="Batch_"&a using the following technique:

Use the following pipetting template: Span-8

Calibration Offset: 0

Calibration Slope: 1.05

Minimum Pipetting Height: 0.5 mm

Prewet: False

Aspirate Blowout: True

Follow Liquid: True

Height: -1.5 mm from the liquid

Mix: True

Mix Aspirate Speed: 100µL/s

Mix Aspirate Height: 1.5 mm from the liquid

Mix Dispense Speed: 400µL/s

Mix Dispense Height: -1.5 mm from the liquid

Mix Count: =nbrOfMix

Mix Volume: 50 µL

Operation speed: 5µL/s

Tip Touch: False

Trailing Air Gap: True

All probes will be used, with a spacing of 1.

The first selected probe will pipette from well =d.

The liquid type is Water and the expected labware type is Plate_96_F_Vis_Greiner.

Span-8 Dispense

Using Pod2, Dispense 0 µL to the labware at ="Batch_"&a using the EHw_Span-8t technique.

All probes will be used, with a spacing of 1.

The first selected probe will pipette to well =d.

Override the technique height by moving to 0 mm from the liquid.

The liquid type is Water and the expected labware type is Plate_96_F_Vis_Greiner.

End Loop

Loop

Loop from "d" = "5" to "11", incrementing by "3".

Span-8 Aspirate

Using Pod2, Aspirate 0 µL from the labware at ="Batch_"&a using the following technique:

Use the following pipetting template: Span-8

Calibration Offset: 0

Calibration Slope: 1.05

Minimum Pipetting Height: 0.5 mm

Prewet: False

Aspirate Blowout: True

Follow Liquid: True

Height: -1.5 mm from the liquid

Mix: True

Mix Aspirate Speed: 100µL/s

Mix Aspirate Height: 1.5 mm from the liquid

Mix Dispense Speed: 400µL/s

Mix Dispense Height: -1.5 mm from the liquid

Mix Count: =nbrOfMix

Mix Volume: 50 µL

Operation speed: 5µL/s

Tip Touch: False

Trailing Air Gap: True

All probes will be used, with a spacing of 1.

The first selected probe will pipette from well =d.

The liquid type is Water and the expected labware type is Plate_96_F_Vis_Greiner.

Span-8 Dispense

Using Pod2, Dispense 0 µL to the labware at ="Batch_"&a using the EHw_Span-8t technique.

All probes will be used, with a spacing of 1.

The first selected probe will pipette to well =d.

Override the technique height by moving to 0 mm from the liquid.

The liquid type is Water and the expected labware type is Plate_96_F_Vis_Greiner.

End Loop

Loop

Loop from "d" = "4" to "10", incrementing by "3".

Span-8 Aspirate

Using Pod2, Aspirate 0 µL from the labware at ="Batch_"&a using the following technique:

Use the following pipetting template: Span-8

Calibration Offset: 0

Calibration Slope: 1.05

Minimum Pipetting Height: 0.5 mm

Prewet: False

Aspirate Blowout: True

Follow Liquid: True

Height: -1.5 mm from the liquid

Mix: True

Mix Aspirate Speed: 100µL/s

Mix Aspirate Height: 1.5 mm from the liquid

Mix Dispense Speed: 400µL/s

Mix Dispense Height: -1.5 mm from the liquid

Mix Count: =nbrOfMix

Mix Volume: 50 µL

Operation speed: 5µL/s

Tip Touch: False

Trailing Air Gap: True

All probes will be used, with a spacing of 1.

The first selected probe will pipette from well =d.

The liquid type is Water and the expected labware type is Plate_96_F_Vis_Greiner.

Span-8 Dispense

Using Pod2, Dispense 0 µL to the labware at ="Batch_"&a using the EHw_Span-8t technique.

All probes will be used, with a spacing of 1.

The first selected probe will pipette to well =d.

Override the technique height by moving to 0 mm from the liquid.

The liquid type is Water and the expected labware type is Plate_96_F_Vis_Greiner.

End Loop

End Group

Span-8 Discard Tips

Discard tips from all probes on Pod2.

End Group

Move Labware

Move the top "1" plates at "="P"&a" to "="P"&a+3" using pod "Pod1".

End Loop

Finish

Method completed.

Remove the tips from all pods. Clear all labware from the deck. Clear all labware from

SILAS devices.Clear all global variables.

REALCAT\Antimicrobial Activities\AntiMAct_Liquid_CMI_Reading_RTU

Method

Author: Beckman Coulter Inc

Description:

Start

FilterMax: Initialize the device, readying it for automated use.

FilterMax: Prepare the device for placement of a piece of labware.

Position: Pelt96_1

Action: Initialize

Instrument Setup

Deck: REALCAT_BKL

Verify that the pod is set up in its default configuration.

Items:

FM1: Nothing

Holder1: Nothing

IN1: Nothing

MID1: Nothing

OUT1: Nothing

P1: Nothing

P10: Nothing

P11: Nothing

P12: Nothing

P13: Nothing

P2: Nothing

P3: Plate_96_F_Vis_Greiner named ReadPlate with known volume: 200 µL of Water in all

wellslid_standard_greiner named Lid_ReadPlate

P4: Nothing

P5: Nothing

P6: Nothing

P7: Nothing

P8: Nothing

P9: Nothing

Pelt96_1: Nothing

PeltFlat_1: Nothing

SPE1: Nothing

TL1: Nothing

TR1: Nothing

W1: Nothing

Move Labware

Move the entire stack of labware at "P3" to "Pelt96_1" using pod "Pod1".

Position: Pelt96_1

Action: Start Shaking

Deluxe Shake?: False

Shake Speed: 900

Shake Style: Orbital (clockwise)

Pause

Pause "the whole system" for "300" seconds.

Position: Pelt96_1

Action: Stop Shaking

Move Labware

Move the entire stack of labware at "Pelt96_1" to "P1" using pod "Pod1".

Move Labware

Move the top "1" plates at "P1" to "P2" using pod "Pod1".

Move Labware

Move the entire stack of labware at "P1" to "FM1" using pod "Pod1".

FilterMax: Run a predefined protocol

REALCAT\Antimicrobial Activities\AntiMAct_Liquid_CMI_Reading_RTU

Move Labware

Move the entire stack of labware at "FM1" to "P3" using pod "Pod1".

Move Labware

Move the entire stack of labware at "P2" to "P3" using pod "Pod1".

Finish

Method completed.

Remove the tips from all pods. Clear all labware from the deck. Clear all labware from

SILAS devices.Clear all global variables.

Figure S7 Liquid handler protocols for the MALDI-Tof target preparation

REALCAT\MALDI\MALDI_96_PCR_Samples+Matrix

12/3/2021 3:52:45 PM Page:1

Method

Author: Beckman Coulter Inc

Description:

Start

Instrument Setup

Deck: REALCAT_BKL

Pause to confirm setup.

Verify that the pod is set up in its default configuration.

Items:

FM1: Nothing

Holder1: Nothing

IN1: Nothing

MID1: Nothing

OUT1: Nothing

P1: Nothing

P10: Nothing

P11: Nothing

P12: Nothing

P13: Nothing

P2: Nothing

P3: Nothing

P4: Nothing

P5: Custom_MALDI_LowProf named Target with known volume: 0 µL of Water in all wells

P6: Nothing

P7: PCR_96_FS_4Titude named Samples with an unknown volume of Water in all

wells.Lid_PCR_4Titude named Lid_Samples

P8: PCR_96_FS_4Titude named Matrix with known volume: 15 µL of Organic in all

wellsLid_PCR_4Titude named Lid_Matrix

P9: Nothing

Pelt96_1: Nothing

PeltFlat_1: Nothing

SPE1: Nothing

TL1: Tips_AP96_20uL named P20. Discard the tips to "<Tipbox>". When done, move the box to

"<Home>". Use these tips "1" times.

TR1: Nothing

W1: Nothing

Group:

Moves

Move Labware

Move the top "1" plates at "P7" to "P4" using pod "Pod1".

Move Labware

Move the top "1" plates at "P8" to "P9" using pod "Pod1".

End Group

Group:

Mixing

New Tips

Load new tips of type "P20" onto pod "Pod1".

Aspirate

Using "Pod1", aspirate "15" µL of "Water" from section 1 of the "PCR_96_FS_4Titude"

labware at "Samples" using the following technique:

Use the following pipetting template: AP96

Calibration Offset: 0

Calibration Slope: 1.04

Minimum Pipetting Height: 0.5 mm

Prewet: False

Aspirate Blowout: False

Follow Liquid: False

Height: 1.5 mm from the bottom

Mix: True

Mix Aspirate Speed: 100µL/s

Mix Aspirate Height: 1.5 mm from the bottom

Mix Dispense Speed: 400µL/s

Mix Dispense Height: 3 mm from the bottom

Mix Count: 10

Mix Volume: 20 µL

Operation speed: 100µL/s

Tip Touch: False

Trailing Air Gap: True

Override the technique height by moving to "1.5" mm from the bottom.

Dispense

Using "Pod1", dispense "15" µL of "Water" to section 1 of the "PCR_96_FS_4Titude" labware

at "Matrix" using the following technique:

Use the following pipetting template: AP96

Calibration Offset: 0

Calibration Slope: 1.04

Minimum Pipetting Height: 0.5 mm

Prewet: True

Blowout: False

Follow Liquid: False

Height: 1.5 mm from the bottom

Mix: True

Mix Aspirate Speed: 75µL/s

Mix Aspirate Height: 1 mm from the bottom

Mix Dispense Speed: 400µL/s

Mix Dispense Height: 3 mm from the bottom

Mix Count: 5

Mix Volume: 10 µL

Operation speed: 10µL/s

Tip Touch: False

Override the technique height by moving to "-1" mm from the liquid.

End Group

Group:

Spoting

Loop

Loop from "a" = "1" to "4", incrementing by "1".

Aspirate

Using "Pod1", aspirate "2" µL of "Organic" from section 1 of the "PCR_96_FS_4Titude"

labware at "Matrix" using (Not Auto-Selected) "EHo_AP96 MALDI" technique.

Dispense

Using "Pod1", dispense "2" µL of "Organic" to sections "=a", of the "Custom_MALDI_LowProf"

labware at "Target" using (Not Auto-Selected) "EHo_AP96 MALDI" technique.

End Loop

Unload Tips

Unload the tips from pod "Pod1".

End Group

Group:

Moves

Move Labware

Move the entire stack of labware at "P9" to "P8" using pod "Pod1".

Move Labware

Move the entire stack of labware at "P4" to "P7" using pod "Pod1".

End Group

Finish

Method completed.

Remove the tips from all pods. Clear all labware from the deck. Clear all labware from

SILAS devices.Clear all global variables.
